# Removal of photoredox catalysts from polymers synthesized by organocatalyzed atom transfer radical polymerization

**DOI:** 10.1002/pol.20220320

**Published:** 2022-07-14

**Authors:** Katherine A. Chism, Daniel A. Corbin, Garret M. Miyake

**Affiliations:** ^1^ Department of Chemistry Colorado State University Fort Collins Colorado USA

**Keywords:** O‐ATRP, photoredox catalyst, polymer purification, radical polymerization

## Abstract

Organocatalyzed atom transfer radical polymerization (O‐ATRP) is a method of producing polymers with precise structures under mild conditions using organic photoredox catalysts (PCs). Due to the unknown toxicity of PCs and their propensity to introduce color in polymers synthesized by this method, removal of the PC from the polymer product can be important for certain applications of polymers produced using O‐ATRP. Current purification methods largely rely on precipitation to remove the PC from the polymer, but a more effective and efficient purification method is needed. In this work, an alternative purification method relying on oxidation of the PC to PC^**·**^^+^ followed by filtration through a plug to remove PC^**·**^^+^ from the polymer and removal of the volatiles was developed. A range of chemical oxidants and stationary phases were tested for their ability to remove PCs from polymers, revealing chemical oxidation by *N*‐bromosuccinimide followed by a filtration through a silica plug can remove up to 99% of the PC from poly(methyl methacrylate). Characterization of the polymer before and after purification demonstrated that polymer molecular weight, dispersity, and chain‐end fidelity are not signficantly impacted by this purification method. Finally, this purification method was tested on a range of dihydrophenazine, phenoxazine, dihydroacridines, and phenothiazine PCs, revealing the strength of the chemical oxidant must match the oxidation potential of the PC for effective purification.

## Graphical abstract

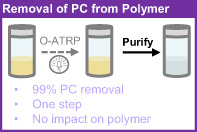

## 1 INTRODUCTION

Organocatalyzed‐atom transfer radical polymerization (O‐ATRP) is a method for producing polymers with precise molecular weights and structures using organic photoredox catalysts (PCs).^1^ Closely related to atom transfer radical polymerization (ATRP),^2^ O‐ATRP employs a similar mechanism to control polymer growth, with the main exception being the use of an organic PC^3^, ^4^ over traditional Cu‐ or Ru‐based catalysts.^5^ As such, O‐ATRP can be more suitable for metal‐sensitive applications^1^ where the use of metal catalysts in ATRP can be problematic.

**FIGURE 1:**
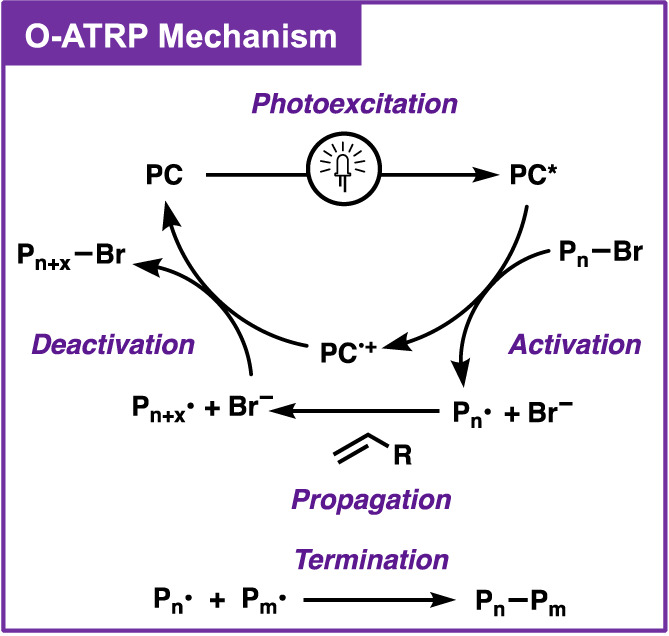
Proposed mechanism of organocatalyzed atom transfer radical polymerization (O‐ATRP) proceeding through an oxidative quenching pathway.

In O‐ATRP, control over the polymer structure is achieved through reversible activation and deactivation of propagating polymer chains (Figure 1).^1^ This process begins with a PC absorbing light to access a highly reducing excited state (PC*). This excited state can then reduce a carbon—halide (often bromide) bond on the initiator or polymer chain‐end in a process called activation. The products of this reaction are a carbon‐centered radical that can begin propagating the polymer chain by reacting with alkene‐based monomers and the radical cation of the PC (PC^**·**^^+^). However, the propagating chains can also react with each other through radical‐based termination reactions, creating dead polymer chains that can no longer be grown by O‐ATRP. To reduce the frequency of termination reactions, a key step in O‐ATRP is deactivation.^6^ During deactivation, the PC^**·**^^+^ reinstalls the bromine chain‐end group on the polymer to create dormant polymer chains. This process lowers the concentration of radicals in solution, therefore decreasing the rate of termination. These dormant polymers can then be reactivated by another PC* to continue propagating until the target molecular weight (*M*_n_) is reached. Therefore, this activation‐deactivation mechanism allows O‐ATRP to produce polymers with targeted molecular weights and low dispersity (*Đ*).

Recent developments in O‐ATRP have focused largely on PC development. When O‐ATRP was first introduced, the photocatalysts reported were perylene^4^ and phenothiazine.^7^ Perylene mediated a moderately controlled polymerization with *Đ* as low as 1.3, and was able to polymerize a range of monomers including methacrylates, acrylates, and styrene.^4^ By contrast, 10‐phenylphenothiazine (PhenS‐Ph) was able to control polymerization with relatively low *Đ* of 1.2.^7^ As such, both perylene and PhenS‐Ph demonstrated potential as O‐ATRP PCs, but improvements were still necessary. Since the excited state of perylene was not as strongly reducing as PhenS‐Ph, more reducing PCs were hypothesized to better activate and control polymerizations by O‐ATRP. However, PhenS‐Ph had to be irradiated by ultraviolet (UV) light, which could cause side reactions within the system, while perylene could operate under visible light. Therefore, strongly reducing excited state PCs that could operate using visible light and exhibit good polymerization control were still needed.

As a result, diaryl dihydrophenazines were developed as PCs that absorb visible light and exhibit strong excited state reduction potentials. *N,N‐*Diaryl dihydrophenazines were introduced with different *N‐*aryl substituents including electron donating groups (EDGs) and electron withdrawing groups (EWG).^8^ While the EDGs related to a stronger PC* reducing state, PCs possessing EWGs provided superior polymerization control.^8^ Shortly thereafter, phenoxazines were introduced that were also capable of absorbing visible light and reaching highly reducing excited states.^9^ This PC family was able to achieve good polymerization control and produce polymers with low *Đ* and achieve high initiator efficiency (I*) using visible light irradiation. Additional photocatalyst families like dihydroacridines were then investigated to try and expand the monomer scope of O‐ATRP.^10^ The aim of the dihydroacridine family was to produce a PC that was highly reducing in the excited state and could quickly deactivate propagating radicals in order to better control polymerizations with a broader range of monomers, especially acrylates.

As a result of these research efforts, the scope of PCs available for O‐ATRP has greatly improved since this method was introduced and the range of O‐ATRP applications has expanded alongside PC development. Due to the organic PCs utilized in O‐ATRP, many applications take advantage of the metal‐free conditions. Specifically, in biological and medicinal applications, the absence of metal has allowed for an expanse of potential uses. Polymers synthesized via O‐ATRP have already been investigated as drug delivery vehicles.^11^ New materials have also been produced using O‐ATRP such as surface‐functionalized diamonds for biological applications^12^ and using O‐ATRP to synthesize methacrylate‐based polymers using monomers derived from biomass feedstocks.^13^ As the scope and control of O‐ATRP continues to expand, so do the potential biological applications.

**FIGURE 2:**
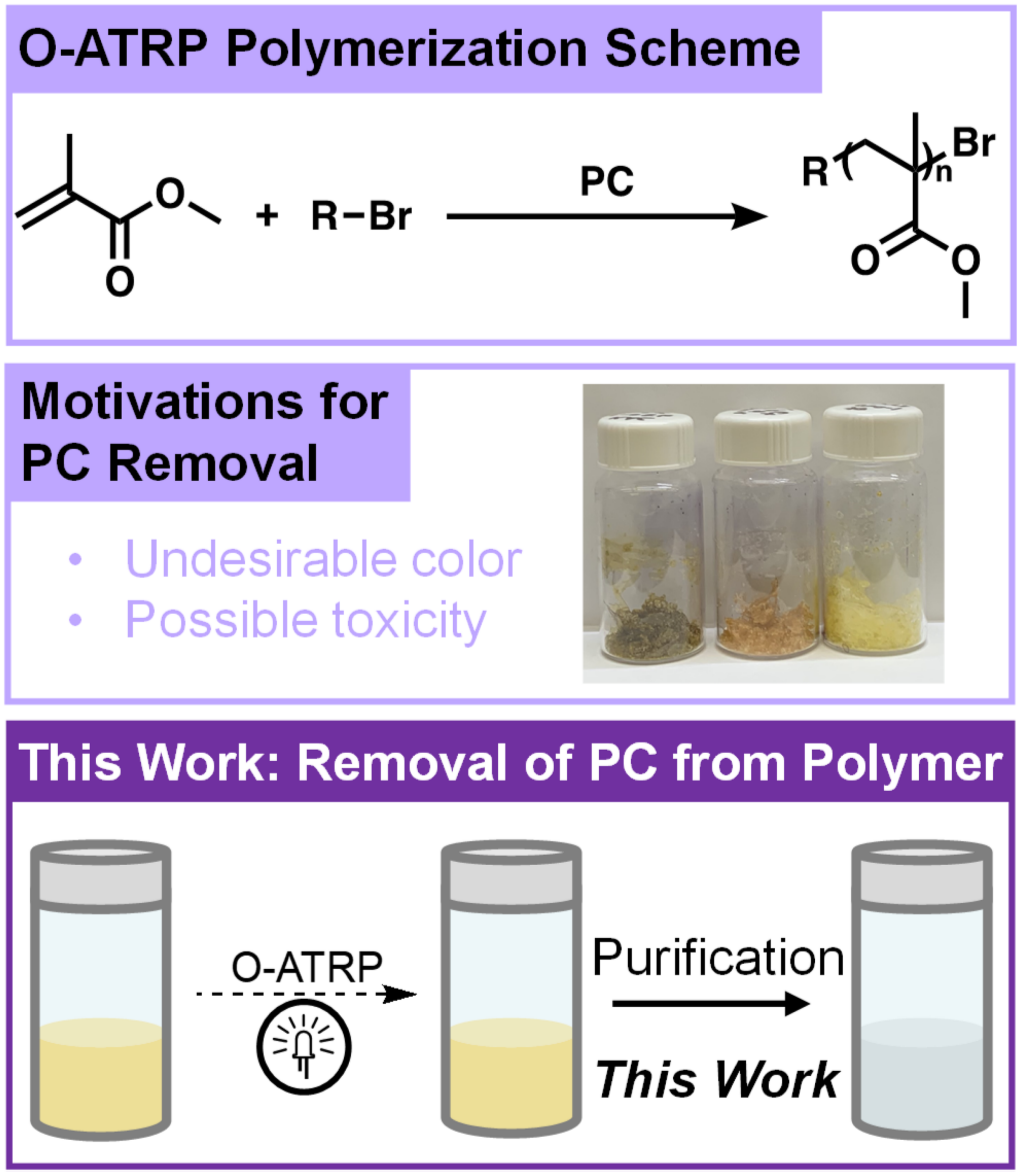
Previous work allowed for the synthesis of poly(methyl methacrylate) (PMMA) using organocatalyzed atom transfer radical polymerization (O‐ATRP) with organic photoredox catalysts (PCs) (top). However, these PCs can impart undesirable color and create toxicity concerns for the product polymers (middle). This work explores the removal of PC from PMMA synthesized by O‐ATRP (bottom).

A problem persists for such applications, however, in that PCs contaminate the synthesized polymer. After O‐ATRP, the PC remains in solution and is often trapped in the polymer matrix upon precipitation commonly used to isolate the polymer. This causes undesirable coloring of the polymer depending on the PC (Figure 2). Additionally, many PCs have unknown toxicities, which questions using polymers synthesized by O‐ATRP in biological applications due to the unknown health effects of the PC. Catalyst purification from polymers produced by O‐ATRP has heretofore been recognized as a tedious process and development of facile and efficient procedures for catalyst removal are required.^14^ Although methods that use low catalyst loadings can minimize catalyst contamination, some applications require complete catalyst removal.^15^, ^16^ As such, a method to remove remaining PC while preserving the synthesized polymer is needed to expand the applications of polymers synthesized by O‐ATRP.

## 2 RESULTS AND DISCUSSION

While several methods exist to remove monomer from polymers synthesized by O‐ATRP, removing the PCs used in these methods has received less focus. Precipitation of the polymer can remove some of the remaining PC by taking advantage of the solubility difference between the polymer and PC. A small amount of PC may also be precipitated out, however, and remains in the collected polymer. As such, this method may not remove all PC from the polymer and often requires several successive precipitations to be performed, making it labor intensive. While investigating the radical cations (PC^·+^) of O‐ATRP PCs,^6^ we noticed these compounds can bind strongly to stationary phases employed in column chromatography such as silica (Figure S10). Based on this observation, we hypothesized this interaction could be used to purify polymers synthesized by O‐ATRP, first by oxidizing the PC to form PC^·+^, followed by a simple filtration through a plug to separate the PC^·+^ from the polymer.

A common PC employed in O‐ATRP is 5,10‐di(4‐trifluoromethyphenyl)‐5,10‐dihydrophenazine (1). In an effort to develop a purification method for O‐ATRP polymers, the ability to remove 1^**·**^^+^ from solution using a plug was first evaluated (see Appendix S1 for more information on the preparation of the plug and a full purification procedure). It was hypothesized 1^·+^ could be separated from the polymer due to the polarity of the stationary phase, which causes a strong interaction with 1^·+^ while allowing the polymer to flow through with relative ease. While several stationary phases were investigated, silica was found to bind 1^·+^ better than alumina or basic alumina (Figure S12). Moreover, binding of 1^·+^ at the top of the silica plug resulted in a clearly visible band of 1^·+^ that was unaffected by the presence of poly(methyl methacrylate) (PMMA) (Figure S10).

To generate 1^·+^, several oxidation methods were explored. It was first envisioned an electrochemical oxidation of 1 to 1^·+^ could be effective, although the 1^·+^ produced by this method could not be separated using silica. No visible band of 1^·+^ was seen on the plug, and the collected solution remained visibly colored (Figure S15). It was hypothesized that the large presence of the supporting electrolyte may have reduced the interaction of 1^·+^ with the stationary phase, leading to higher mobility of 1^·+^ in the plug. To avoid the presence of the supporting electrolyte, chemical oxidation with Br_2_, I_2_, HNO_3_, a Br_2_·dioxane complex, and *N*‐bromosuccinimide (NBS) was investigated. In each case, 1^·+^ successfully formed a distinct band at the top of the plug with all tested oxidants (see Appendix S1).

**FIGURE 3:**
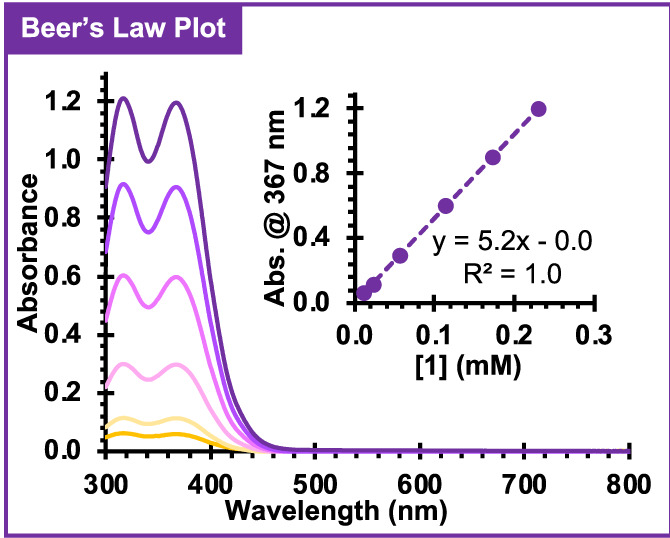
Absorption spectra of 1 at various concentrations in *N,N*‐dimethylacetamide with poly(methyl methacrylate) present. Absorbance values at 367 nm were used to construct the Beer's Law plot (inset), giving a molar absorptivity of 5200 cm^−1^ M^−1^.

A Beer's Law plot of 1 was created to quantify the amount of 1 present in the polymer by measuring the absorbance of 1 in *N,N*‐dimethylacetamide (DMAc) at various concentrations. The absorbance of 1 showed maximum absorption peaks at 317 and 367 nm (Figure 3), and the peak at 367 nm was chosen to evaluate the [1] in each sample. At this wavelength, a molar absorptivity of 5200 cm^−1^ M^−1^ was calculated, which is in agreement with previously reported values.^15^ In addition, the absorbance of 1 was not affected by the presence of PMMA, as the same molar absorptivity of 1 was obtained in the presence and absence of PMMA (Figure S6).

To investigate the removal of PCs from polymers synthesized by O‐ATRP, PC 1 was used to synthesize PMMA under previously reported conditions.^8^, ^17^ Large scale O‐ATRP polymerizations often suffer from non‐uniform irradiation and produce poorly defined polymers,^18^ so multiple small‐scale polymerizations were performed to maintain optimal polymerization control. Two blended batches of PMMA were synthesized by O‐ATRP using PC 1, DBMM, and methyl methacrylate (MMA) (1:10:1000 eq, respectively), and the blended polymers were characterized before and after purification (see Appendix S1 *)*. The amount of 1 g of PMMA in each batch was quantified before and after purification to evaluate the efficacy of each purification method. Prior to purification, batch 1 contained 7.3 mg of 1 g of PMMA, and batch 2 contained 8.0 mg of 1 g of PMMA (Table 1).

**TABLE 1:** PC concentration after purification and percent PC removed during purification

Method	Initial amt. of 1 (mg/g PMMA)^a^	Final amt. of 1 (mg/g PMMA)^b^	1 removed (%)	PMMA recovered (%)^c^
First precipitation	7.3	3.2	56	—
Second precipitation	7.3	1.4	81	—
Third precipitation	7.3	0.5	93	63^e^
Br_2_	8.0	0.2	98^d^	69
I_2_	8.0	1.0	88	71
HNO_3_	8.0	0.2	98	66
Br_2_·dioxane	8.0	0.2	98	57^f^
NBS	8.0	0.1	99	79

Abbreviations: NBS, *N*‐bromosuccinimide; PC, photoredox catalysts; PMMA, poly(methyl methacrylate).

^a^ Determined based on the absorbance of 1 prior to polymer purification.

^b^ Determined based on the absorbance of 1 after polymer purification.

^c^ Unless otherwise noted, purification was performed starting with 300 mg PMMA.

^d^ Denotes a maximum percent recovery. Samples after each precipitation were set aside for analysis, so more polymer would likely be lost if the entire batch underwent three precipitations.

^e^ Average of three samples.

^f^ Purification started with 100 mg PMMA.

**FIGURE 4:**
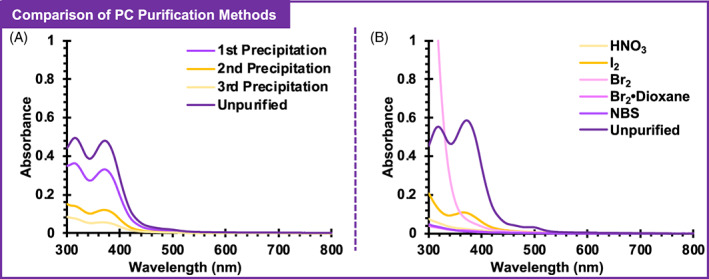
UV‐visible absorption spectra of poly(methyl methacrylate) synthesized using 1 before and after purification by successive precipitation (A) or chemical oxidation followed by a silica plug (B). Chemical oxidants are specified in the figure legend.

To determine if purification with a silica plug was more efficient than existing methods, it was compared to purification by precipitation.^15^, ^19^ In precipitation, the mixture of PMMA and PC is dissolved in dichloromethane (DCM) and the PMMA is slowly precipitated into a cold anti‐solvent (methanol), see Appendix S1 for the exact precipitation procedure used in this work. The difference in solubility between the polymer and PC allows some PC to remain in solution as PMMA precipitates, thus increasing the purity of the polymer. After a single precipitation, 56% of initial 1 present was removed, and a portion of PMMA was set aside for analysis. The remaining PMMA was then precipitated a second time, and a portion was set aside. These two successive precipitations removed 81% of initial 1 present. Finally, the remaining PMMA was purified a third time. After three successive precipitations, 93% of 1 had been removed from the polymer (Table 1). The absorption spectra of each PMMA sample purified by precipitation contained visible peaks for 1 (Figure 4), further highlighting the inefficiency of purifying these polymers by this precipitation process.

While precipitation did not appear to be a very efficient method of purification, it was unclear if chemical oxidation followed by a silica plug would result in improved purification. To explore this possibility, chemical oxidation with Br_2_, I_2_, HNO_3_, a Br_2_·dioxane complex, and NBS was investigated. These oxidants ranged in standard reduction potential (E^0^), with Br_2_ being the strongest oxidant (See Appendix S1). Since quantitative addition of Br_2_ can be challenging, the use of a Br_2_·dioxane complex allowed for a more quantitative addition of Br_2_ in a 1:1 ratio with 1, since this complex is a solid. I_2_, HNO_3_, and NBS were tested to evaluate the efficiency of oxidants with lower E^0^ values.

The percent of 1 removed by chemical oxidation and filtration though a silica plug ranged from 88% to 99%. Oxidation with I_2_ removed the least amount of 1 (88%) and oxidation with NBS removed the greatest amount of 1 (99%). In addition, Br_2_, I_2_, and HNO_3_ all required quenching and further purification after removal of 1, which took additional time and caused shoulders to appear in the absorbance spectra of these samples. Purification with Br_2_ specifically caused a broad, red‐shifted shoulder as a result of quenching, although no peaks corresponding to 1 were observed (Figure 4). In fact, many of the absorbance spectra of purified PMMA showed no visible peaks for 1 (Figure 4), suggesting most, if not all, of 1 had been removed from these samples.

**FIGURE 5:**
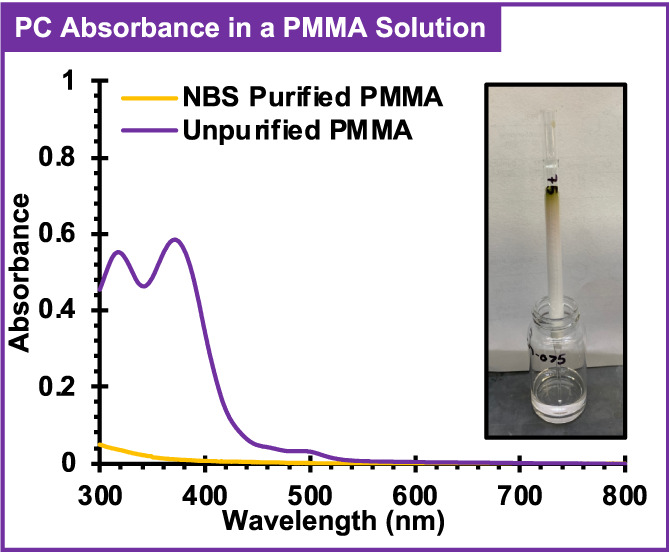
UV‐visible absorption spectrum of poly(methyl methacrylate) (PMMA) synthesized with 1 before (purple) and after purification (yellow) by chemical oxidation with *N*‐bromosuccinimide (NBS) and purification using a silica plug. Figure inset shows a photograph of the silica plug and purified PMMA solution. See Appendix S1 for purification details.

In perhaps the best system, chemical oxidation with NBS and filtration using a silica plug resulted in the collection of a visibly transparent and colorless solution, the absorption spectrum of which showed no peaks corresponding to 1 (Figure 5). As an added benefit, NBS did not require quenching as was the case for other chemical oxidants, since NBS is also removed by the silica plug.^20^ Analysis by ^1^H NMR confirmed the removal of excess NBS from purified polymer samples (Figure S28). As such, chemical oxidation by NBS and removal of the resulting 1^·+^ by a silica plug was demonstrated as an effective way to separate 1 from PMMA.

In addition to removing higher amounts of 1, this new purification method required less work and resulted in greater recovery of the desired polymer product. Successive precipitation required over 5 hrs of work, whereas chemical oxidation and a silica plug could be completed within 1 h. Additionally, precipitation resulted in lower percent recovery than chemical oxidation. After three successive precipitations, a maximum of 63% recovery was observed (Table 1). The percent recovery for chemical oxidation followed by a silica plug ranged from 57% to 79% for each of the chemical oxidants (Table 1). However, it should be noted the percent recovery of 57% was obtained starting with 100 mg PMMA, whereas all other purification experiments were performed starting with 300 mg PMMA. For these larger samples, percent recovery ranged from 66% to 79%. In other words, the new purification method developed in this work generally resulted in greater recovery of the desired polymer, better removal of 1 from the polymer, and greater time efficiency by eliminating the need for successive purification steps.

While this new purification method appeared superior to precipitation, the potential effects of chemical oxidants on the polymer structure remained to be investigated. To evaluate if any of these purification methods alter polymer structure, polymers were characterized before and after purification by gel permeation chromatography (GPC) to investigate impacts on molecular weight (*M*_n_), dispersity (*Đ*), and chain‐end fidelity. In each case, polymers were characterized in triplicate over three separate days to determine an average and standard deviation for *M*_n_ and *Đ*. The *M*_n_ values were determined to be 9.55 ± 0.4 kDa and 8.95 ± 0.5 kDa for PMMA batch 1 and 2, respectively. After purification, no significant change was observed in either *M*_n_ or *Đ*. The *M*_n_ after precipitation ranged from 9.87 kDa to 10.7 kDa in comparison to the 9.55 kDa prior to purification (Table 2). However, considering the standard deviations for these measurements, these deviations do not appear significant. The same is true for all the chemical oxidants, as no major changes in *M*_n_ were observed as a result of purification. In these cases, *M*_n_ ranged from 9.69 kDa to 11.0 kDa in comparison to the 8.95 kDa initial *M*_n,_ although these deviations also appear insignificant when considering the standard deviation of these values.

**TABLE 2:** Characterization of PMMA before and after purification.

Method	Initial *M*_n_ (kDa)^a^ ^,^ ^b^ ^,^ ^c^	Final *M*_n_ (kDa)^b^ ^,^ ^c^	Initial *Đ* ^a^ ^,^ ^b^ ^,^ ^c^	Final *Đ* ^b^ ^,^ ^c^
First precipitation	9.55 ± 0.37	10.3 ± 0.3	1.10 ± 0.04	1.06 ± 0.02
Second precipitation	9.55 ± 0.37	9.87 ± 0.81	1.10 ± 0.04	1.08 ± 0.04
Third precipitation	9.55 ± 0.37	10.7 ± 0.7	1.10 ± 0.04	1.07 ± 0.04
Br_2_	8.95 ± 0.54	10.1 ± 0.4	1.08 ± 0.02	1.07 ± 0.02
I_2_	8.95 ± 0.54	9.69 ± 0.44	1.08 ± 0.02	1.06 ± 0.02
HNO_3_	8.95 ± 0.54	9.71 ± 0.50	1.08 ± 0.02	1.06 ± 0.03
Br_2_·dioxane	8.95 ± 0.54	11.0 ± 0.5	1.08 ± 0.02	1.06 ± 0.05
NBS	8.95 ± 0.54	9.84 ± 0.62	1.08 ± 0.02	1.06 ± 0.02

Abbreviations: GPC, gel permeation chromatography; NBS, *N*‐bromosuccinimide; PMMA, poly(methyl methacrylate); THF, tetrahydrofuran.

^a^ Averaged values from multiple samples of blended PMMA synthesized using 1 (see Appendix S1 for more information on polymer synthesis and blending).

^b^ Determined by gel permeation chromatography coupled with multi‐angle light scattering in THF.

^c^ Averaged values and standard deviation across samples run on three separate days.

With respect to *Đ*, this parameter was also relatively consistent before and after purification for all tested methods. After precipitation, *Đ* decreased slightly for all purified PMMA samples (Table 2). A lower *Đ* could indicate a loss of lower molecular weight chains during precipitation due to variations in solubility with polymer chain length. However, these small decreases in *Đ* are within the standard deviation in *Đ* prior to purification. For samples purified by chemical oxidation and a silica plug, even smaller variations in *Đ* were observed ranging from 1.06 to 1.08 for all samples before and after purification.

To evaluate possible effects on chain‐end fidelity, chain extension polymerizations were performed comparing unpurified PMMA versus purified PMMA as macro‐initiators in O‐ATRP. In a chain extension experiment, previously synthesized polymers with bromine chain‐end groups can be used as initiators to begin propagation of additional monomer units. As a result, an increase in polymer molecular weight should be observable, indicating the presence of these bromine chain‐end groups. Instead, polymers without this bromine chain‐end group should be unable to continue propagation, resulting in no change in their molecular weight. As such, the ability of the purified polymer to act as a macro‐initiator can indicate impacts on chain‐end group fidelity. If chain‐end group fidelity was unaltered during purification, it was predicted that the unpurified and purified PMMA samples would show similar chain‐extended GPC traces.

**FIGURE 6:**
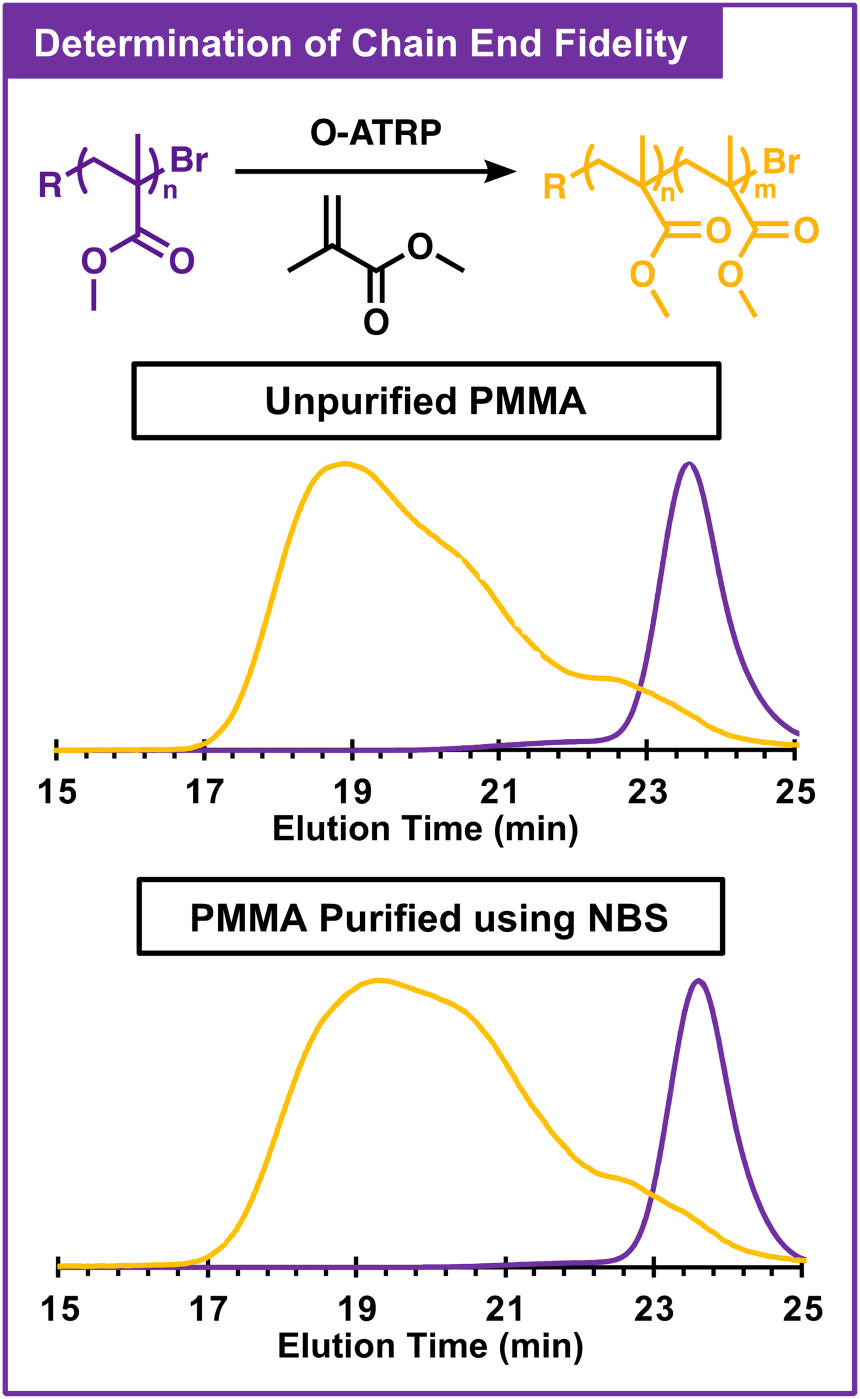
Gel permeation chromatography traces before (purple) and after (yellow) chain extension of poly(methyl methacrylate) (PMMA) that was used as a macro‐initiator in organocatalyzed atom transfer radical polymerization (O‐ATRP) without purification (top), as well as after purification with *N*‐bromosuccinimide (NBS) followed by a silica plug to remove residual 1 (bottom). Traces shown were collected with a differential refractive index detector (for more information and associated light scattering traces, see Appendix S1).

Of all the purification methods tested, only the Br_2_ system appeared to alter chain‐end group fidelity and resulted in a significantly altered chain extension trace relative to unpurified PMMA (Figure S30). A similar trace was seen for unpurified and purified PMMA using all other oxidants. In particular, PMMA purified using NBS showed a very similar trace to the unpurified sample, indicating good retention of chain‐end fidelity during purification by this method (Figure 6).

**FIGURE 7:**
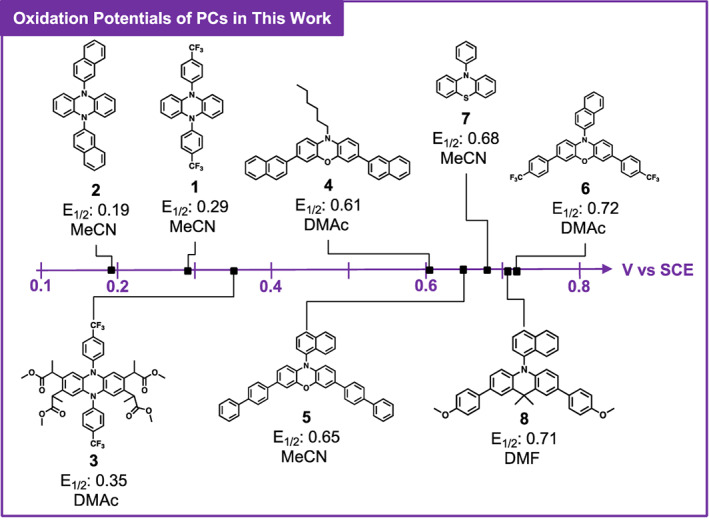
Reported oxidation potentials^7^, ^8^, ^9^, ^10^, ^17^, ^21^, ^22^ measured by cyclic voltammetry for photoredox catalysts (PCs) used to synthesize poly(methyl methacrylate) via organocatalyzed atom transfer radical polymerization. Solvent used in cyclic voltammetry is indicated under E_1/2_ values.

After determining oxidation with NBS and purification with a silica plug removed the most percent PC and did not alter *M*_n_, *Đ*, or chain‐end group fidelity, the range of photocatalysts that could be removed by this method remained to be investigated. A range of phenoxazines, dihydrophenazines, dihydroacridines, and phenothiazines were selected to be oxidized and removed from PMMA (Figure 7). Notably, the PCs chosen range in their oxidation potentials [E^0^ (PC^·+^/PC) ~ E_1/2_] from 0.19 V versus saturated calomel electrode (SCE) to 0.72 V versus SCE, allowing the impact of PC oxidation potential on purification efficacy to be investigated. PMMA was synthesized with each PC using O‐ATRP, and the same purification method developed for 1 was followed (see Appendix S1 *Section Purification of PMMA Synthesized with Other PCs*).

Interestingly, oxidation with NBS and silica plug purification worked better with less oxidizing PCs. For samples synthesized using PCs 1, 2, and 3, comparison of the absorbance spectra before and after purification revealed most of the PC was successfully removed. For PCs 1, 2, and 3, the estimated percent PC removal ranged from 89% to 96% (Table 3). For samples prepared with PCs 2 and 3, there appeared to be no PC peaks present (Figures S37 and S38), although a slight PC peak for 1 remained in UV‐visible (Figure S36). The presence of this residual PC is likely a result of having to estimate the quantity of 1 present in the unpurified polymer, which may have resulted in the addition of sub‐stoichiometric quantities of NBS and incomplete PC oxidation. For PC 4, an estimated 22% PC was removed during purification. It should be noted there was a significant increase in E_1/2_ between 3 and 4, from 0.35 V to 0.61 V versus SCE (Table 3). As such, NBS may not have been sufficiently oxidizing to convert all of 4 to 4^**·**^^+^.

**TABLE 3:** Impact of PC oxidation potential on polymer purification with NBS.

PC	Ref.	*E*_1/2_ ^a^ ^,^ ^b^ (V vs. SCE)	% PC removed^e^
1	8	0.29^c^	89
2	8	0.19^c^	96
3	17	0.35	94
4	21	0.61	22
5	9	0.65^c^	0
6	22	0.72	4
7	7	0.68^c^	18
8	10	0.71^d^	17

Abbreviations: MeCN, methyl cyanide; DMAc, *N*,*N*‐dimethylacetamide; DMF, dimethylformamide; NBS, *N*‐bromosuccinimide; PC, photoredox catalysts; PMMA, poly(methyl methacrylate); SCE, saturated calomel electrode.

^a^ *E*_1/2_ ~ *E* ^0^(PC^+^/PC).

^b^ Unless otherwise noted, *E*_1/2_ values were measured in DMAc by cyclic voltammetry.

^c^ *E*_1/2_ measured in MeCN.

^d^ *E*_1/2_ measured in DMF.

^e^ % PC removed = (*A*_before_ – *A*_after_)/*A*_before_ × 100% (see Appendix S1).

For samples prepared with PCs 5, 6, 7, and 8, little‐to‐no PC appeared to be removed during purification (Table 3). The estimated percent PC removal ranged from 0% to 18% for these more oxidizing PCs. Absorbance values for these samples were very similar before and after purification (Figures S40–S43), again indicating NBS may not be sufficiently oxidizing to convert these PCs to PC^·+^ for purification. While the silica plugs for 5, 6, 7, and 8 displayed brightly colored bands as expected, it is possible NBS may have only oxidized a small portion of the PC present, although not enough to effectively purify the polymer. For PCs with higher E_1/2_ values such as these, using a stronger oxidant would most likely improve purification. Previously, a similar effect was observed with oxidation of 1 with I_2_, where this relatively weak oxidant resulted in poor PC removal but was improved upon using stronger oxidants. As such, for PCs 5, 6, 7, and 8, stronger oxidants such as Br_2_ or the Br_2_˙dioxane complex could be better alternatives.

## 3 CONCLUSION

This work investigated the potential of chemical oxidation of the PC followed by filtration using a silica plug as a new and improved method for polymer purification following O‐ATRP. The amount of PC present in a polymer sample was quantified using UV‐visible absorption spectroscopy to quantify the amount of PC per gram of PMMA before and after purification. When purification was achieved by three successive precipitations, 93% of 1 was removed from PMMA and 62.77% of the polymer was recovered, although this process was extremely labor intensive. By contrast, chemical oxidation with NBS followed by filtration using a silica plug was found to remove 99% of 1 from PMMA in a single step while enabling 78.69% of the PMMA to be recovered. Key to the superior performance of this method was the ability of 1^·+^ to bind strongly to silica, even in the presence of PMMA. Further, several chemical oxidants can be used to oxidize the PC, including Br_2_, I_2_, HNO_3_, a Br_2_·dioxane complex, and NBS.

To understand whether this purification method impacts polymer structure, *M*_n_, *Đ*, and chain‐end fidelity were investigated before and after purification for each method. No changes in *M*_n_ and *Đ* were observed before and after purification for all investigated methods. Chain‐end fidelity also appeared unaltered for most of the methods investigated in this work, although excess Br_2_ was observed to have detrimental impacts on this property.

The scope of PCs that could be oxidized by NBS and removed with a silica plug was then explored. PCs from various families including phenoxazines, dihydrophenazines, dihydroacridines, and phenothiazines were investigated. These experiments revealed that purification success depends on the E^0^ (PC^·+^/PC) of the PC and the strength of the oxidant. In other words, strongly oxidizing PCs require stronger chemical oxidants to convert the PC to PC^·+^ for successful purification. As such, an appropriate oxidant must be chosen depending on the PC being removed. Overall, chemical oxidation followed by a silica plug was found to be a successful method of purification that did not alter the synthesized polymer. We envision this work will be crucial to the implementation of polymers synthesized by O‐ATRP in biological applications and applications where the color imparted by O‐ATRP PCs is undesirable.

## AUTHOR CONTRIBUTIONS

**Garret M. Miyake:** Conceptualization (supporting); project administration (lead); supervision (lead); writing – review and editing (supporting). **Katherine A. Chism:** Conceptualization (equal); data curation (lead); formal analysis (lead); writing – original draft (lead); writing – review and editing (lead). **Daniel A. Corbin:** Conceptualization (equal); data curation (equal); formal analysis (equal); writing – original draft (supporting); writing – review and editing (supporting).

## Supporting information


**Appendix S1** Supporting Information.
