# Supplementary material for: Removal of photoredox catalysts from polymers synthesized by organocatalyzed atom transfer radical polymerization
Source: J Polym Sci (2020). 2022 Jul 14;60(19):2747–55. doi: 10.1002/pol.20220320 (PMC9796344; doi:10.1002/pol.20220320)
Supplement: Supplementary file 1 — Appendix S1 Supporting Information. [file POLA-60-2747-s001.pdf]

Supporting Information
for

Removal of Photoredox Catalysts from Polymers Synthesized by Organocatalyzed Atom Transfer Radical Polymerization

Katherine A. Chism, Daniel A. Corbin, Garret M. Miyake*

Department of Chemistry, Colorado State University, 200 W. Lake St., Fort Collins, Colorado 80523, United States

*Email: garret.miyake@colostate.edu

Table of Contents

Materials and Methods 4
Purchased Chemicals 4
Chemical Preparation and Storage 4
Experimental Equipment 4
Procedures 6
Synthesis of Photocatalysts 6
Synthesis of Blended PMMA 7
Polymer Purification by Precipitation 9
Polymer Purification by Chemical Oxidation and Silica Plug 9
Determination of [1] in PMMA by UV-Vis 9
UV Vis 10
Absorbance of 1 with and without PMMA 10
Absorbance of PMMA at Various Concentrations 11
Initial [1] in PMMA 12
Preliminary Investigation of $1^{•+}$ Separation using Plugs 13
Electrochemical Oxidation of 1 to $1^{•+}$ 16
Purification by Precipitation 18
Chemical Oxidants and Silica Plugs of $1^{•+}$ 19
Br_2 Oxidation of 1 and Silica Plug Purification 19
I_2 Oxidation of 1 and Silica Plug Purification 20
HNO_3 Oxidation of 1 and Silica Plug Purification 21
Br_2 · Dioxane Oxidation of 1 and Silica Plug Purification 22
NBS Oxidation of 1 and Silica Plug Purification 23
Chain Extension 24
General Chain Extension Procedure 24
Unpurified PMMA 25
Br_2 Purified PMMA 25
I_2 Purified PMMA 25
HNO_3 Purified PMMA 26
Br_2 · dioxane Purified PMMA 26
NBS Purified PMMA 27

Oxidation Potential of Chemical Oxidants *28*
Cyclic Voltammetry of NBS 28
Oxidation Potentials of Additional Chemical Oxidants 28
Purification of PMMA Synthesized with Other PCs *29*
O-ATRP Synthesis of PMMA with PCs 2-8 29
General PC Purification Scheme 29
UV-Vis Before and After Purification 29
References *34*

Materials and Methods

Purchased Chemicals

For the synthesis of 1: Sodium tert-butoxide, 2-dicyclohexylphosphino-2'-6'-diisopropoxybiphenyl (RuPhos), RuPhos Pd G4, 4-bromobenzotrifluoride, and dioxane were all purchased from Sigma Aldrich.

For the synthesis of 2-8: Chemical compounds used to synthesize these photoredox catalysts (PCs) were reported in previously published papers.[1-7]

For O-ATRP: Methyl methacrylate (MMA), diethyl-2-bromo-2-methylmalonate (DBMM), ethyl acetate (EtAc), and *N,N*-dimethylacetamide (DMAc) were all purchased from Sigma Aldrich.

For electrochemistry: Tetra-n-butylammonium hexafluorophosphate (NBu_4PF_6), silver nitrate, and DCM were purchased from Sigma Aldrich. Acetonitrile was purchased from Fisher Scientific.

For chemical oxidation: Molecular bromine and *N*-bromosuccinimide (NBS) were purchased from Sigma Aldrich. Molecular iodine was purchased from Alfa Aesar. Nitric acid was purchased from Millipore.

For purification: DCM and alumina were purchased from Sigma Aldrich. Sodium hydroxide and silica were purchased from Fisher Scientific. Basic alumina and sodium thiosulfate were purchased from Acros Organics.

For UV-Vis: DMAc was purchased from Sigma Aldrich.

For free radical polymerization of PMMA: MMA and AIBN were both purchased from Sigma Aldrich.

Chemical Preparation and Storage

For PCs: PCs were stored in vials open to air and light once fully synthesized.

For O-ATRP: MMA and DBMM were dried using calcium hydride, vacuum distilled, freeze-pump-thawed to remove oxygen, and stored under nitrogen in the dark and in a freezer at -40ºC. They were warmed to room temperature prior to use. EtAc and DMAc were anhydrous and stored under nitrogen.

For PMMA Synthesized via O-ATRP: Synthesized PMMA was removed from its solvent and vacuum dried at 70 ºC. The PMMA was then stored at room temperature exposed to oxygen.

Experimental Equipment

Light Reactors for O-ATRP: For light beakers, white LEDs and UV light strips were used. A 400 mL beaker (10.0 cm tall, 8.5 cm diameter) was wrapped in aluminum foil and duct taped. LED strips were then wrapped along the inside (9 LED segments, 16" total) of the beaker (Figure S1). White LED strips were purchased from Creative Lighting Solutions (item no. CL-FRS1210-5M-12V-WH). UV light strips 380-385 nm were purchased from LEDlightinghut (LED type 300 pcs SMD5050).

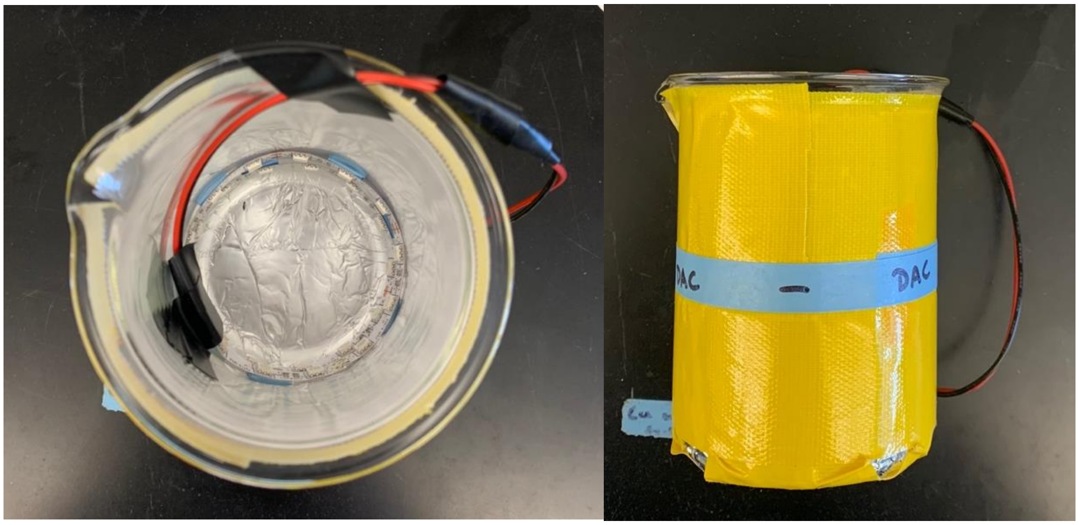

Figure S1. Representative figure of the light beakers used for O-ATRP showing top view (left) and side view (right).

Electrochemistry: For electrochemistry performed in this work, 0.1 M Bu_4NPF_6 was the supporting electrolyte. Cyclic voltammetry was performed in a three-electrode cell. Glassy carbon was the working electrode; platinum was the counter electrode, and silver/silver nitrate (0.01 M $AgNO_3$ in MeCN with 0.1 M Bu_4NPF_6) was the reference electrode. Potentials were adjusted to be vs saturated calomel electrode (SCE) by adding 0.29 V to the potential vs $AgNO_3$.

Instrumentation

Nuclear magnetic resonance (NMR) spectroscopy was performed with a Bruker US 400 MHZ spectrometer. All ^{1}H NMR spectra are reported in δ units, parts per million (ppm) and are referenced to the residual NMR solvent signal. To determine molecular weights of the polymers, gel permeation chromatograph (GPC) coupled with multi-angle light scattering (MALS) using an Agilent HPLC fitted with one guard column, three PLgel 5 µm MIXED-C gel permeation columns, a Wyatt Technology TrEX differential refractometer, and a Wyatt Technology miniDAWN TREOS light scattering detector was used with THF as the eluent at a flow rate of 1.0 mL/min. A dn/dc value of 0.084 was used for PMMA. Electrochemical measurements were performed using a Gamry Interface 1010E potentiostat. UV-Visible spectroscopy was performed using an Agilent Cary 5000 UV-Vis-NIR spectrometer.

Procedures

<u>*Synthesis of Photocatalysts*</u>

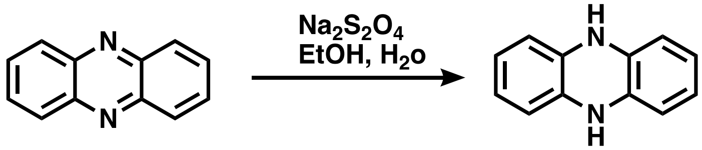

Figure S2. Scheme of 5, 10-dihydrophenazine synthesis

Synthesis of 5,10-dihydrophenazine: Dihydrophenazine was synthesized according to a previously reported literature procedure.[1] ^{1}H NMR characterization matched previously reported data for this compound.

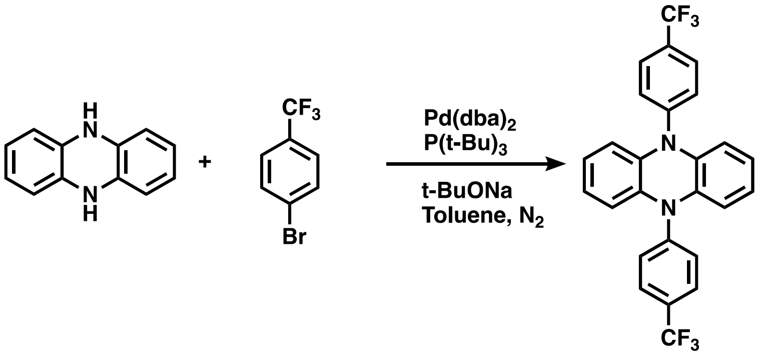

Figure S3. Scheme of PhenN-PhCF_3 (**1**) Synthesis

Synthesis of 5,10-di(4-trifluoromethylphenyl)-5,10-dihydrophenazine (1): PC **1** was synthesized according to a previously reported literature procedure.[1] ^{1}H NMR characterization matched previously reported data for this compound.

Synthesis of 5,10-di(2-naphthyl)-5,10-dihydrophenazine (2): PC **2** was synthesized according to a published literature procedure.[1]

Synthesis of 2,3,7,8-tetra(methylpropionoate-yl)-5,10-di(4-trifluoromethylphenyl)-5,10-dihydrophenazine (3): PC **3** was synthesized according to a published literature procedure.[2]

Synthesis of 3,7-bis(2-napthalenyl)-1-hexyl-10-phenoxazine (4): PC **4** was synthesized according to a published literature procedure.[3]

Synthesis of 3.7-di(4-biphenyl)-1-naphthalene-10-phenoxazine (5): PC **5** was synthesized according to a published literature procedure.[4]

Synthesis of 3,7-di(4-(4-trifluoromethyl)phenyl)-2-napthalene-10-phenoxazine (6): PC **6** was synthesized according to a published literature procedure.[5]

Synthesis of 10-phenylphenothiazine (7): PC **7** was synthesized according to a published literature procedure.[6]

Synthesis of 2,7-bis(4-methoxyphenyl)-9,9-dimethyl-10-(napthalen-1-yl)-9,10-dihydroacridine (8): PC **8** was synthesized according to a published literature procedure.[7]

Synthesis of Blended PMMA

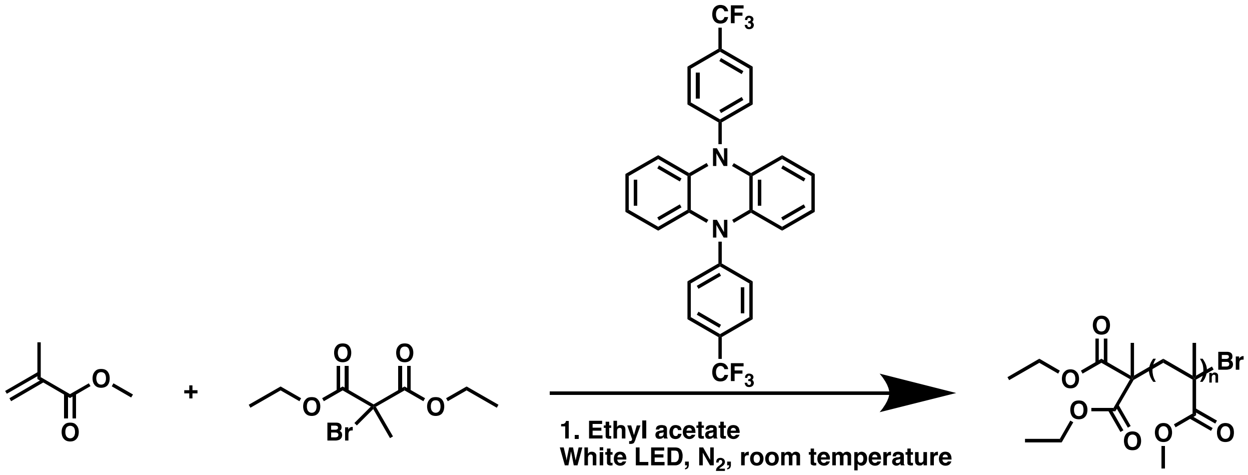

Figure S4. Scheme of PMMA Synthesis

Synthesis of PMMA: 1 (0.0094 mmol, 1 eq) was weighed into six 20 mL scintillation vials (Table S1) and brought into a nitrogen filled glovebox. Ethyl acetate (1.00 mL) was added to each vial. In the dark, MMA (1.00 mL, 9.35 mmol, 1000 eq) and DBMM (17.9 µL, 0.0935 mmol, 10 eq) were added to each vial. The reactions were then stirred and irradiated for 24 hours in white LED beakers. A desktop fan was used for temperature control. White LED beakers had been warmed up (~30 minutes) before vials were added to ensure consistent and uniform irradiation. After 24 hours, an 0.1 mL aliquot of vial A was added to a solution of $CDCl_3$ with 250 ppm BHT and analyzed by ^1H NMR to evaluate percent conversion. The vials were then exposed to oxygen and dried under forced air. This process was repeated for another 6 times on a separate day using the same methods and stoichiometry above. An aliquot of vial G was taken after 24 hours. All 12 samples were then dried in a vacuum oven at 70°C for 72 hours.

Table S1. Amount of **1** used in synthesis of PMMA

**Vial**	**Amount of** 1 **(mg)**
**A**	5.0
**B**	4.7
**C**	4.1
**D**	4.5
**E**	4.7
**F**	4.3
**G**	5.0
**H**	5.0
**I**	4.4
**J**	4.2
**K**	5.1
**L**	4.3

Blending of PMMA Batches 1 and 2: Two batches of blended PMMA were created by combining vials A-F (batch 1) and G-L (batch 2) respectively. The blended PMMA was then characterized to determine mg **1** g^{-1} PMMA, M_n, and *Đ*.

Synthesis of PMMA by Free Radical Polymerization

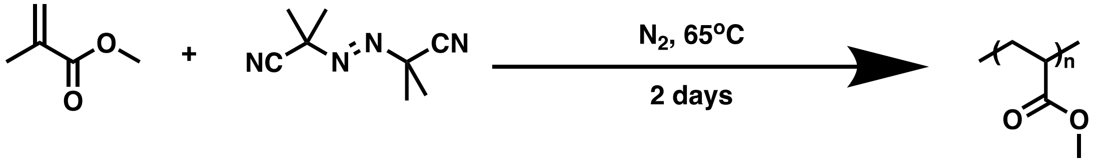

Figure S5. Scheme of PMMA vis free radical polymerization

Synthesis of PMMA by Free Radical Polymerization (FRP): AIBN (0.413 mmol, 1 eq) was weighed into a vial and MMA (93.89 mmol, 227 eq) was added to the vial with a stir bar. The solution was sparged for 20 minutes under nitrogen. Solution was then heated at 65ºC under nitrogen while stirring for 2 days. Vial was then cooled to room temperature and transferred to a larger flask. It was rotovapped at 35ºC and dried under vacuum at 70ºC for 4 days to dry.

Synthesis of Bromine Dioxane Complex

Synthesis of Br_2 • dioxane: The bromine dioxane complex was synthesized by following a published literature procedure.[8]

Polymer Purification by Precipitation

Purification by precipitation: 50.0 mL of methanol was stirred so a vortex formed in a flask at -78ºC. 804.9 mg of unpurified PMMA (Batch 1) was then dissolved in a minimal amount of DCM (5.00 mL). The PMMA dissolved in DCM solution was then added dropwise to the methanol using a syringe and 0.45 µm syringe filter. The solid precipitate was then collected by vacuum filtration. After the first precipitation, 155.2 mg of purified PMMA was set aside for analysis. The remaining purified PMMA was redissolved in a minimal amount of DCM, and precipitated into methanol repeating the procedure listed above. After the second precipitation, 106.0 mg of PMMA was set aside, and the remaining PMMA was precipitated a third time. After the third precipitation, 244.0 mg of PMMA was collected.

All three precipitation purified samples were dried under a fan overnight and then dried under vacuum at 70ºC for 24 hours.

Polymer Purification by Chemical Oxidation and Silica Plug

Chemical Oxidation: Unless otherwise noted, 300.0 mg of PMMA was dissolved in 6.00 mL of DCM for chemical oxidations. The estimated amount of PC per g PMMA was used to determine equivalence with oxidants. Oxidants were added to dissolved PMMA while stirring. For exact equivalencies for each oxidant see *Chemical Oxidants and Silica Plugs of* ***1***$^{\cdot+}$ in *Supporting Information.* Solutions were then allowed to stir for 10 minutes before silica plug purification.

Silica Plug Purification: A silica plug composed of a Pasteur pipette with a chem wipe at the bottom and 800.0 mg of silica was used for purification. The oxidized PC and PMMA solution was passed through a silica plug and collected. Air was forced through the plug for efficiency. Plugs were rinsed with 2.00 mL of DCM after all PMMA solution was passed through. Oxidants that required quenching were quenched 3 times and the organic layer was extracted (See *Chemical Oxidants and Silica Plugs of* ***1***$^{\cdot+}$ in *Supporting Information*). Collected purified solutions were dried overnight under a fan. They were then dried for 2 days under vacuum at 70ºC.

*Determination of [**1**] in PMMA by UV-Vis*

Determination of [1]: Purified PMMA was dissolved in DCM in a desired density of 6.00 mg/mL for purified complexes. In PC scope investigations PMMA in DCM densities were targeted 3.00 mg/mL.

UV Vis

*<u>Absorbance of **1** with and without PMMA</u>*

Trial	Molar Absorptivity (cm^{-1} M^{-1})
PMMA	5400
No PMMA	5400

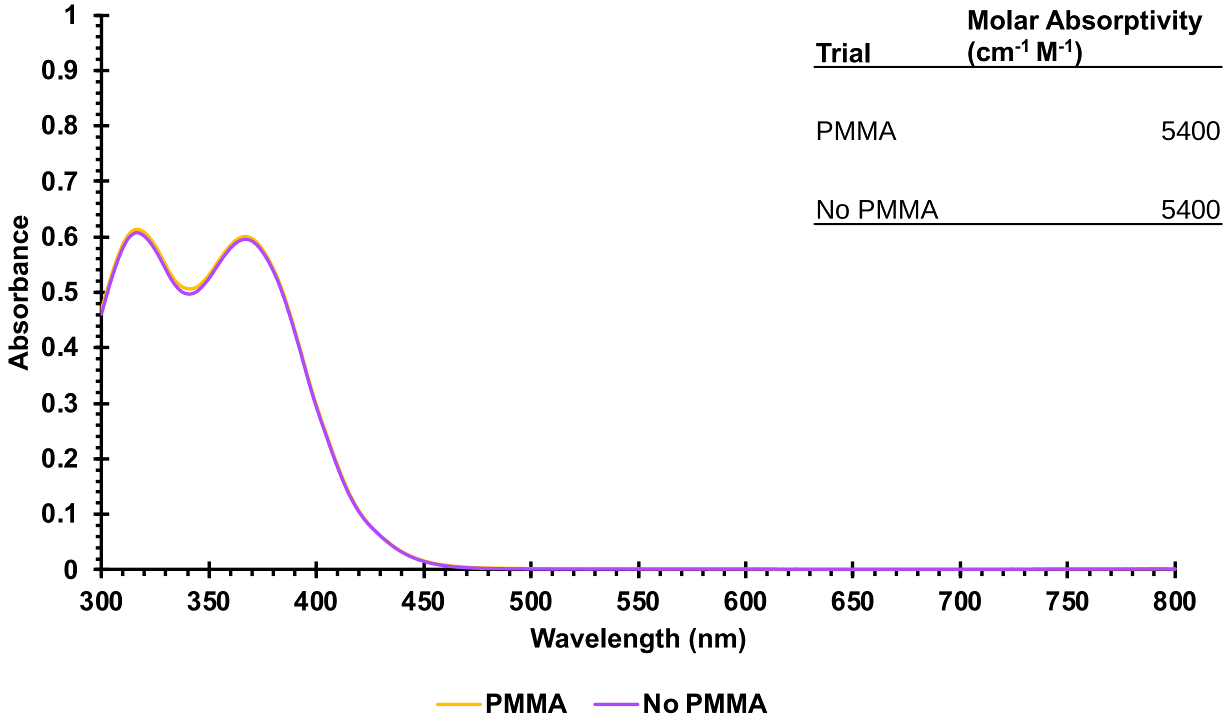

Figure S6. UV-Vis absorbance spectrum for 0.111 mM **1** in DMAc. One DMAc solution contained 6.24 mg/mL of PMMA (yellow), and the other solution contained only DMAc (purple). Presence of PMMA was shown not to impact UV-Vis spectra or absorbance.

No change in molar absorptivity was seen for **1** in the presence or absence of PMMA, indicating PMMA should not affect absorbance values (Figure S6).

Absorbance of PMMA at Various Concentrations

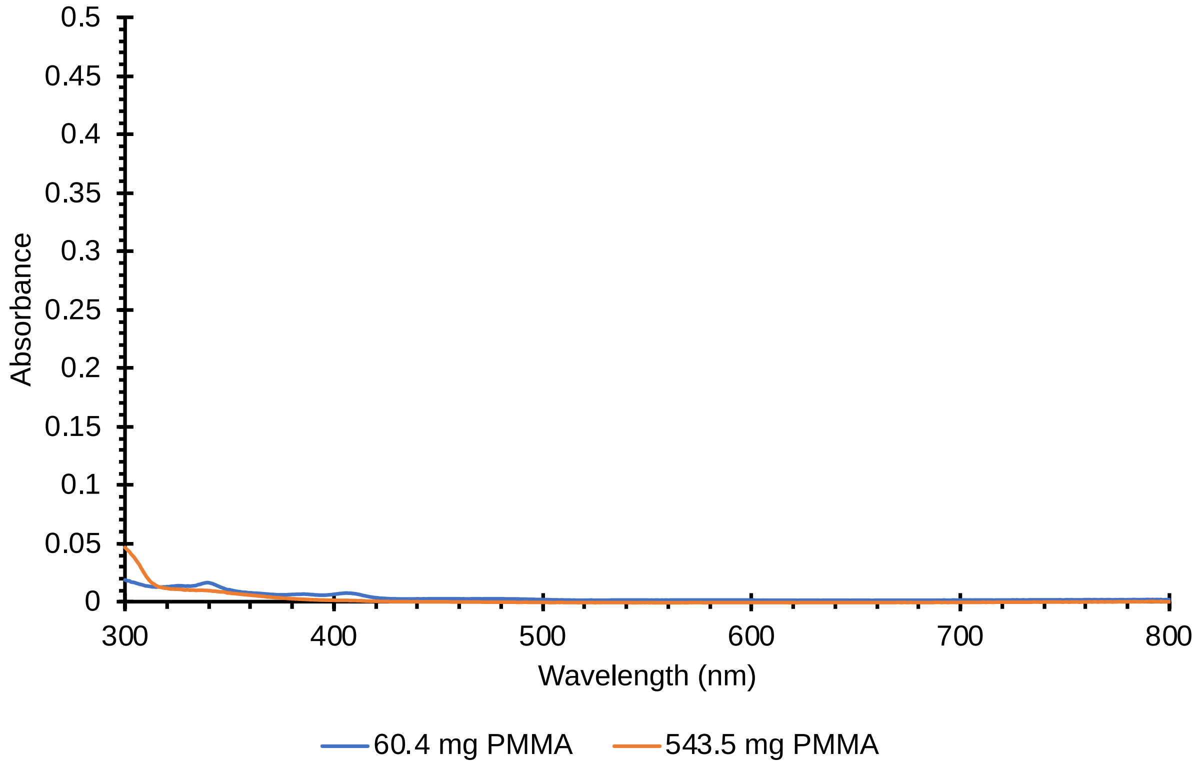

Figure S7. UV-Vis Absorbance of PMMA in DMAc with one solution containing 6.04 mg/mL PMMA in DMAc (blue) and the other containing 54.35 mg/mL PMMA in DMAc (orange). A baseline of DMAc was used.

No significant change in absorbance was observed between the two concentrations indicating concentration of PMMA in DMAc should not impact absorbance values (Figure S7).

Initial [1] in PMMA

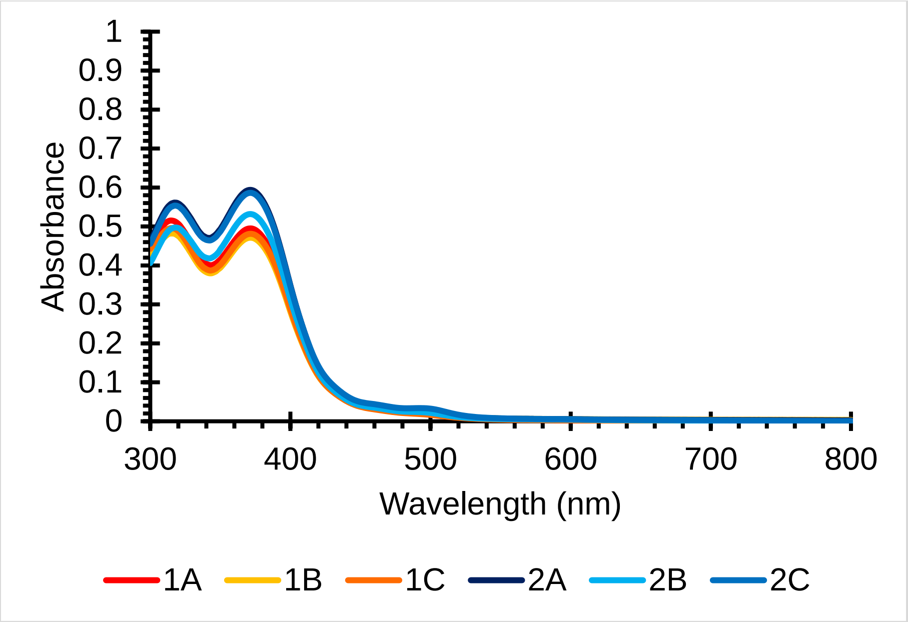

Figure S8. Absorbance values of batch 1 and batch 2 blended PMMA. 3 trials were conducted for each and averaged.

To determine the amount of **1** per gram of PMMA, three samples of ~60 mg PMMA dissolved in DMAc (10.00 mL) were prepared, dissolved, and evaluated by UV-Vis spectroscopy (Table S2). Using the Beer's Law plot and molar absorptivity the average amount of **1** per gram of PMMA was determined. Absorbance spectra are shown in figure S8. Individual trial values of mg **1** · g^{-1} PMMA (Table S2), and average values and standard deviation (Table S3).

Table S2. Determination of mg **1** · g^{-1} PMMA for blended PMMA batches

Trial	Mass of PMMA (g)	Absorbance (@367 nm)	[1] (mM)	mmole 1	Amount 1 (mg)	mg 1 · g^{-1} PMMA
**1A**	0.0604	0.490	0.094	0.00094	0.44	7.3
**1B**	0.0573	0.466	0.090	0.00090	0.42	7.4
**1C**	0.0589	0.475	0.091	0.00091	0.43	7.3
**2A**	0.0672	0.587	0.11	0.0011	0.53	7.9
**2B**	0.0594	0.526	0.10	0.0010	0.48	8.0
**2C**	0.0650	0.580	0.11	0.0011	0.52	8.1

Table S3. Computed average of mg **1** per g PMMA for PMMA batches 1 and 2.

Batch	Avg mg 1 · g^{-1} PMMA	Std Dev
**1**	7.3	0.03
**2**	8.0	0.08

Preliminary Investigation of $1^{\bullet+}$ Separation using Plugs

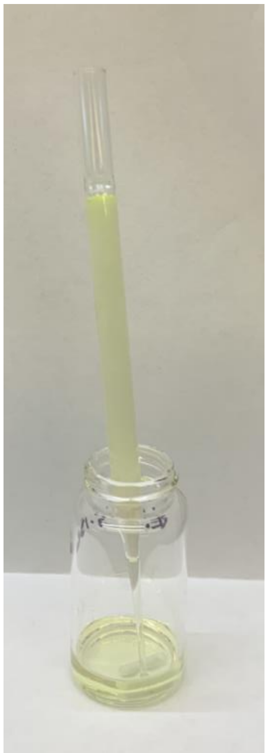

Figure S9. Photograph depicting silica plug capture of **1** in DCM.

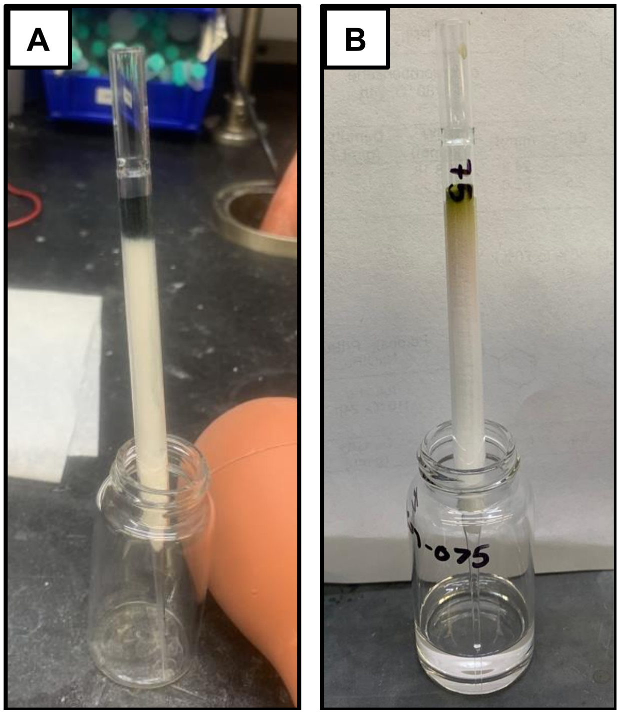

Figure S10. Photographs depicting silica plug capture of $\mathbf{1}^{\bullet+}$ in DCM (A) and DCM with PMMA (B).

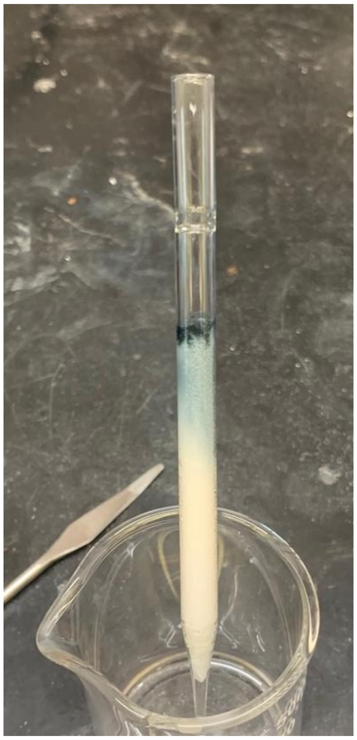

Figure S11. Photograph depicting basic alumina plug capture of **$1^{\bullet+}$** in DCM.

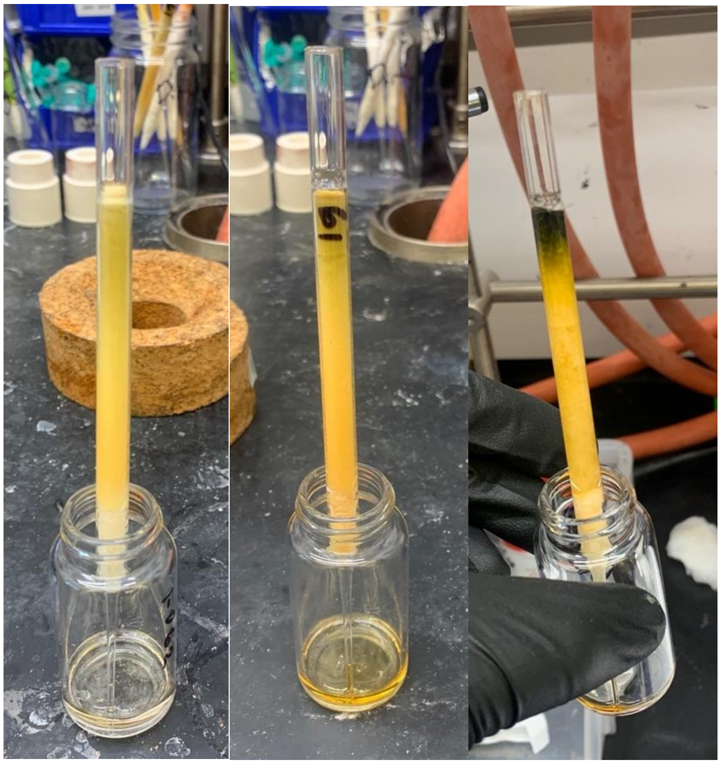

Figure S12. Basic alumina (left), alumina (middle), silica (right) plugs of Br_2 oxidized **$1^{\bullet+}$** and PMMA.

1 was found not to stick to silica (Figure S9). $\mathbf{1}^{\bullet+}$ was seen to run down the alumina and basic alumina plugs (Figure S11). $\mathbf{1}^{\bullet+}$ was found to stick best to silica when compared to alumina and basic alumina (Figure S12). The ability of $\mathbf{1}^{\bullet+}$ to stick to silica was found be unaffected by the presence of PMMA (Figure S10).

Electrochemical Oxidation of 1 to 1$^{\bullet+}$

Cyclic Voltammetry: A solution of 2 mM **1** was prepared in DCM with 0.1 M Bu_4NPF_6. The solution was analyzed by cyclic voltammetry (CV) using a glassy carbon working electrode, a platinum counter electrode, and a silver/silver nitrate reference electrode (0.01 M $AgNO_3$ in acetonitrile with 0.1 M Bu_4NPF_6). Prior to measurement, the solution was sparged with N_2 for 10 minutes.

This was repeated twice with a solution of 2 mM **1** (Figure S13) and a solution of 237.0 mg PMMA containing **1** (estimated 1.9 mg, 0.004 mmol, 0.4 mM **1**) (Figure S14).

Bulk Electrolysis: The solution from CV was then oxidized by bulk electrolysis. A glassy carbon plate was used as the working electrode. The solution was stirred and run at a 400 mV applied potential until roughly 0 A of current (about 3 hours). This experiment was performed with a solution of only **1** as well as a solution of PMMA (~ 250 mg) with **1** (~ 2 mg).

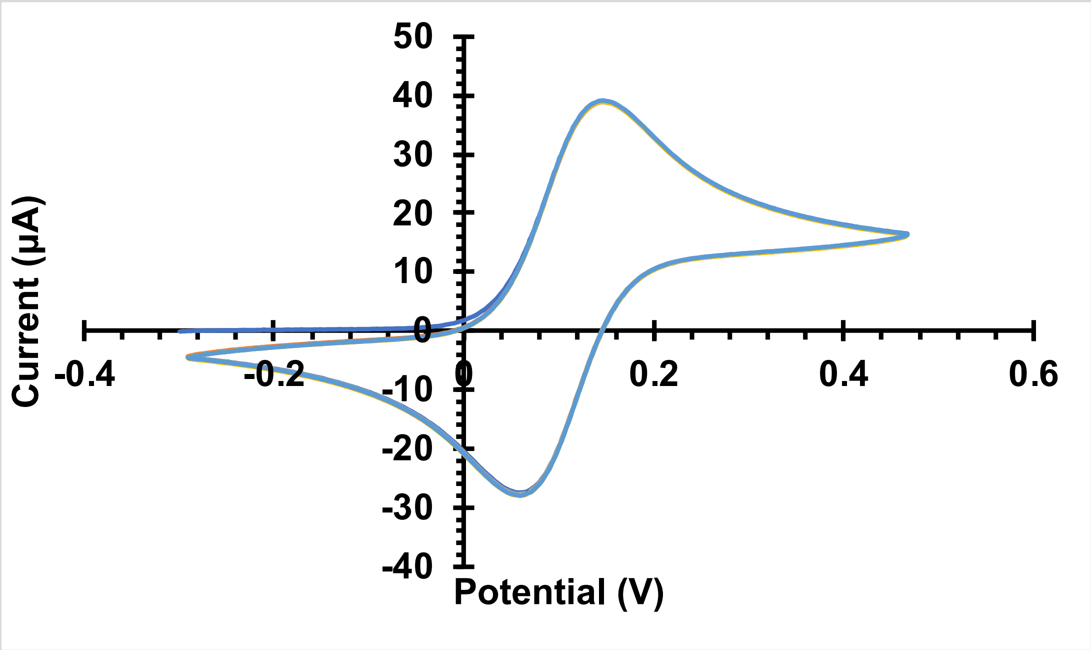

Figure S13. Cyclic Voltammogram of **1** (2 mM) scanning from low to high potentials (-0.3 V to 0.5 V) in DCM.

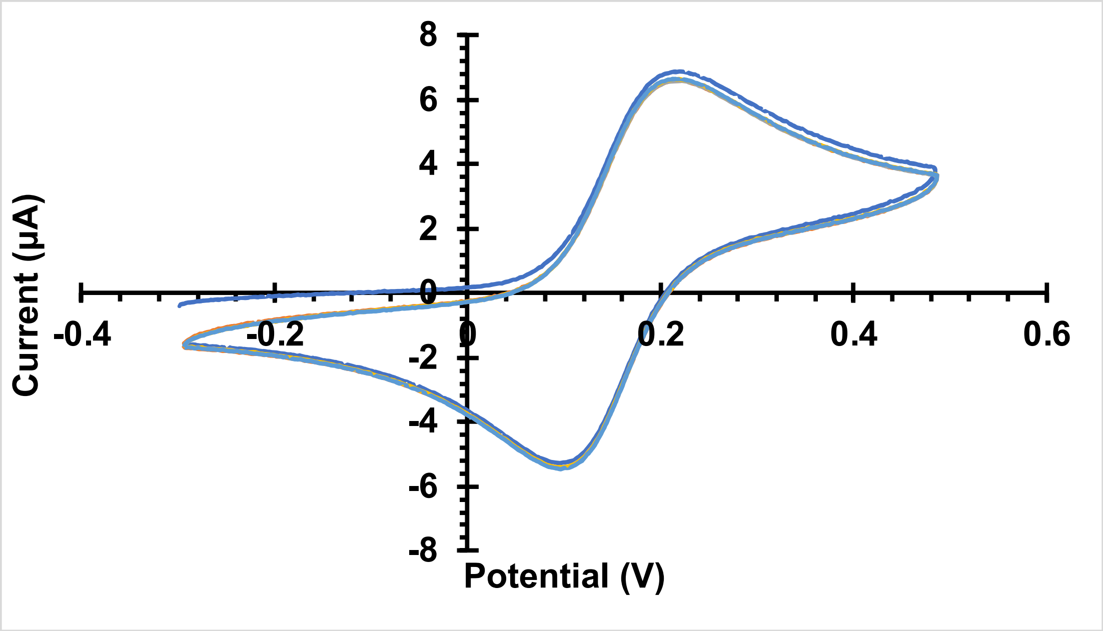

Figure S14. Cyclic Voltammogram of **1** (estimated 0.4 mM) in the presence of PMMA (237.0 mg) scanning from low to high potentials (-0.3 V to 0.5 V) in DCM.

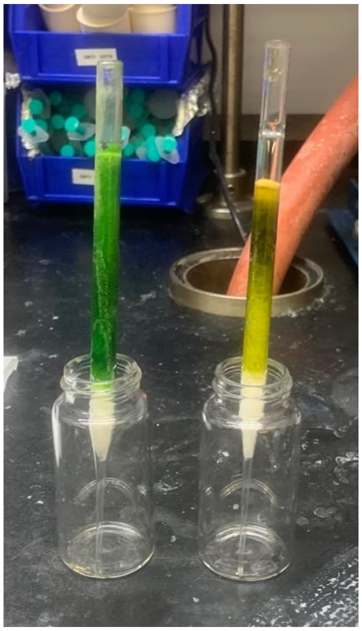

Figure S15. Silica plug capture of **1**$^{•+}$ oxidized by electrochemistry depicting bulk electrolysis of only **1**$^{•+}$ (left) and bulk electrolysis of PMMA and **1**$^{•+}$ (right).

Purification by Precipitation

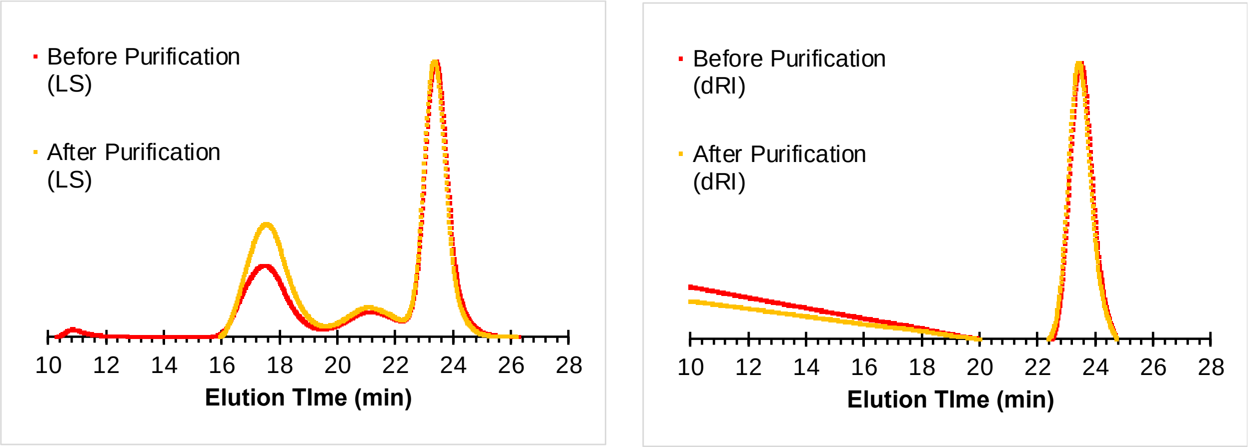

Figure S16. Purified PMMA after one precipitation before purification (red) and after purification (orange) using gel permeation chromatography light scattering (left) and dRI (right).

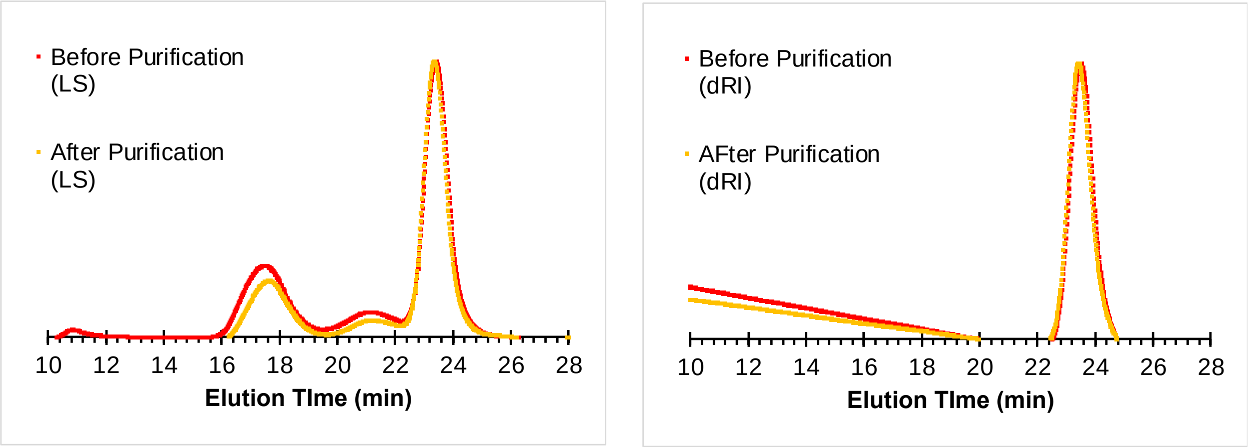

Figure S17. Purified PMMA after two precipitations before purification (red) and after purification (orange) using gel permeation chromatography light scattering (left) and dRI (right).

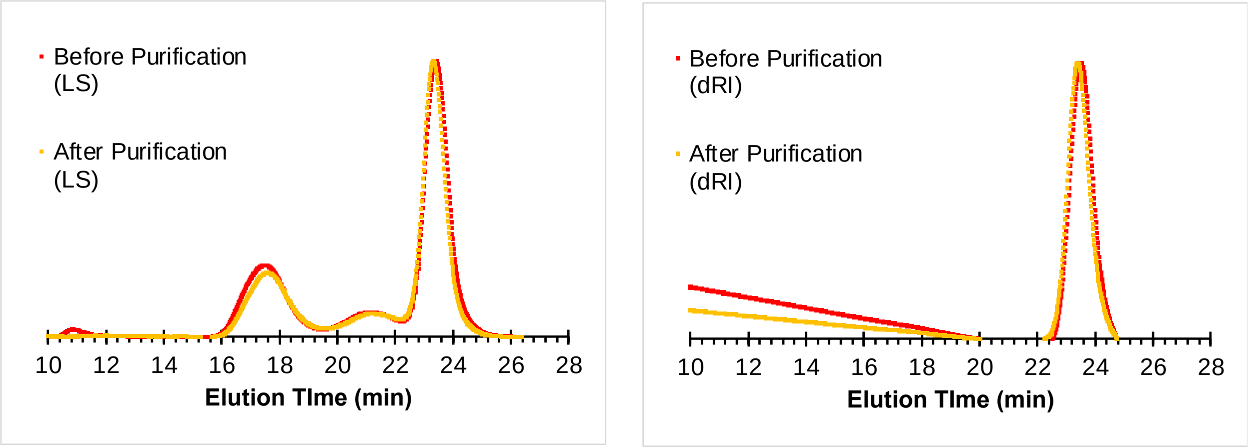

Figure S18. Purified PMMA after three precipitations before purification (red) and after purification (orange) using gel permeation chromatography light scattering (left) and dRI (right).

Chemical Oxidants and Silica Plugs of $\mathbf{1}^{\bullet+}$

<u>*Br_2 Oxidation of **1** and Silica Plug Purification*</u>

Br_2 oxidation: 300.0 mg of PMMA containing residual **1** from O-ATRP was dissolved in 6.00 mL DCM. Solution was stirred until all PMMA dissolved. A 1.00 mL plastic syringe and 22 G needle was used to add 3 drops of Br_2 (excess) to the PMMA solution while stirring. Solution was allowed to stir 10 minutes and turned dark purple/brown in color. Solution was then purified with a silica plug (See *Polymer Purification by Chemical Oxidation and Silica Plug* in *Supporting Information*). The collected solution was quenched with a saturated $Na_2S_2O_3$ aqueous solution. A separatory funnel was used to separate the organic and aqueous layers (~10 mL each). Separation was repeated 3 times and the organic layer was collected each time. Collected PMMA was dried under fan and then under vacuum at 70°C.

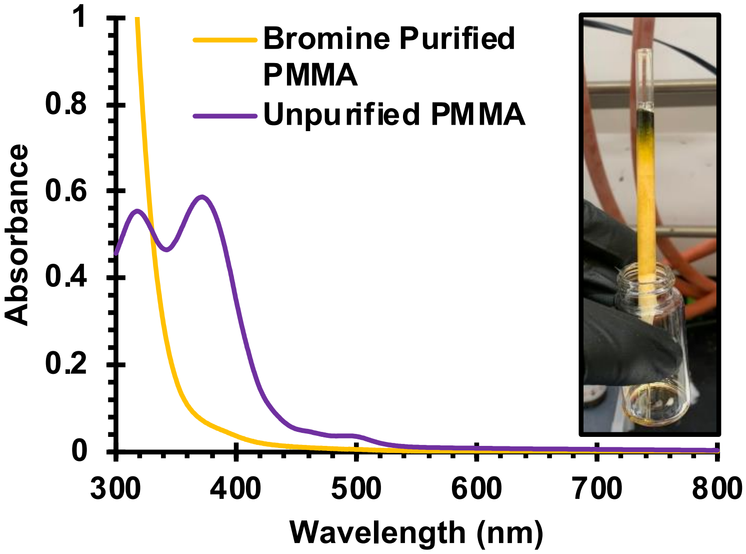

Figure S19. Br_2 oxidized $\mathbf{1}^{\bullet+}$ and silica plug purification. A broad shoulder was seen on all bromine purifications, but no PC peak at 367 nm was visible.

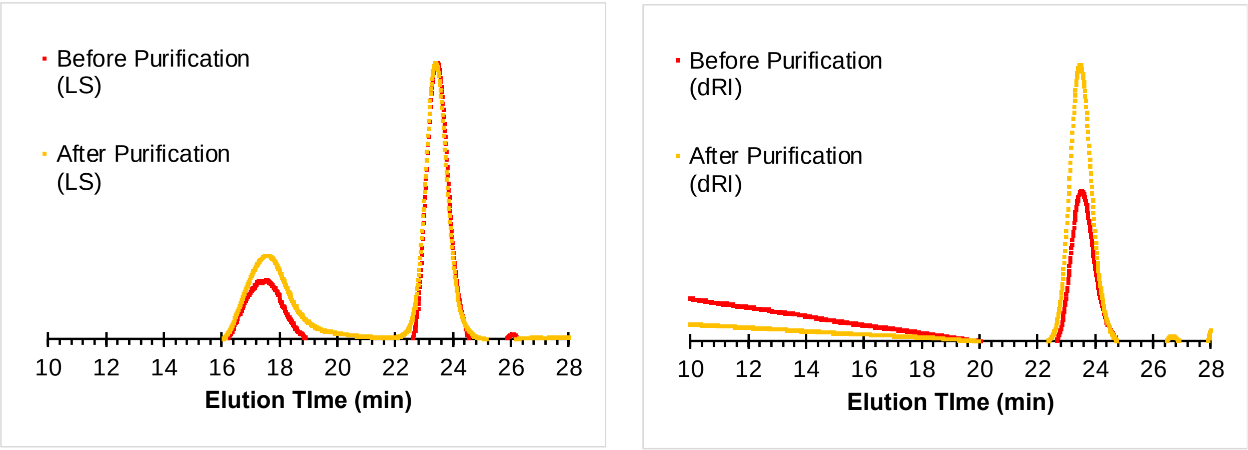

Figure S20. Br_2 oxidized and silica plug purified PMMA before purification (red) and after purification (orange) using gel permeation chromatography light scattering (left) and dRI (right).

*I_2 Oxidation of **1** and Silica Plug Purification*

I_2 oxidation: 300.0 mg of PMMA containing residual **1** from O-ATRP was dissolved in 6.00 mL DCM. Solution was stirred until all PMMA dissolved. I_2 (8.7 mg, 5 eq, 0.035 mmol) was added the PMMA solution while stirring. Equivalence was determined by the estimation of PC remaining in PMMA (8.0 mg **1** • g^{-1} PMMA). Solution was allowed to stir 10 minutes and turned dark red/black in color. The solution was then purified with a silica plug (See *Polymer Purification by Chemical Oxidation and Silica Plug* in *Supporting Information*). The collected solution was quenched with a saturated $Na_2S_2O_3$ aqueous solution. A separatory funnel was used to separate the organic and aqueous layers (~10 mL each). Separation was repeated 3 times and the organic layer was collected each time. Collected PMMA was dried under fan and then under vacuum at 70°C.

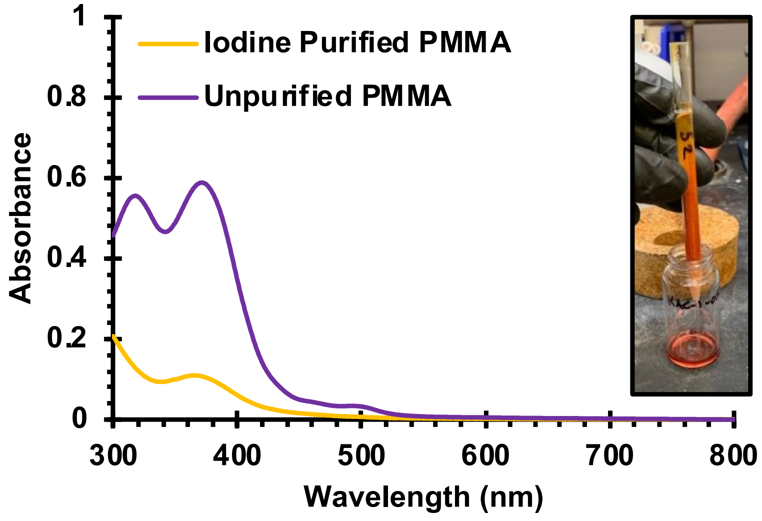

Figure S21. I_2 oxidized $\mathbf{1}^{\bullet+}$ and silica plug purification. A PC peak at 367 nm was still visible.

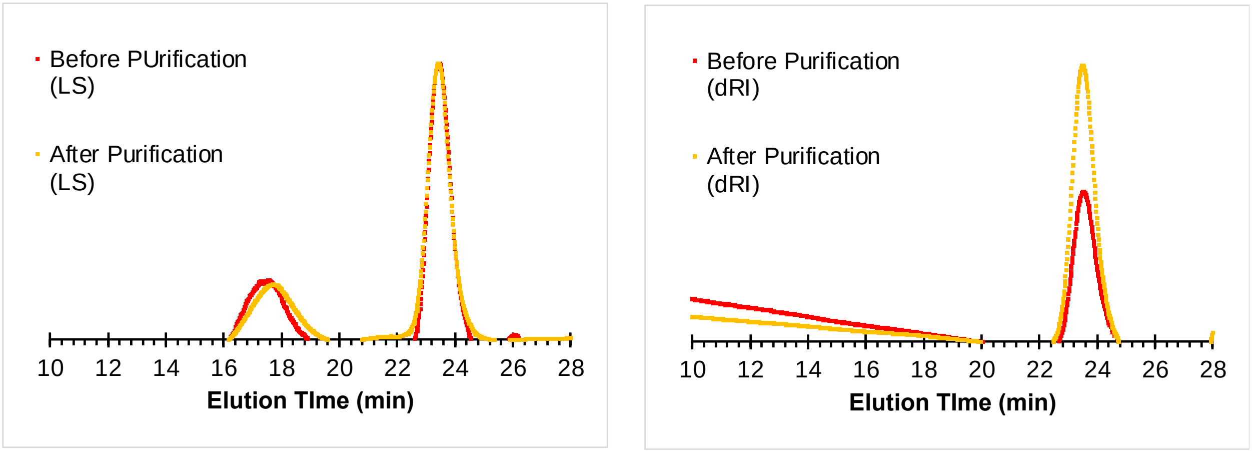

Figure S22. I_2 oxidized and silica plug purified PMMA before purification (red) and after purification (orange) using gel permeation chromatography light scattering (left) and dRI (right).

*HNO_3 Oxidation of **1** and Silica Plug Purification*

HNO_3 oxidation: 300.0 mg of PMMA containing residual **1** from O-ATRP was dissolved in 6.00 mL DCM. Solution was stirred until all PMMA dissolved. A 1.00 mL plastic syringe and 22 G needle was used to add 3 drops of 1 M HNO_3 (excess) to the PMMA solution while stirring. The solution was allowed to stir 10 minutes, and no color change was observed. Solution was then purified with a silica plug (See *Polymer Purification by Chemical Oxidation and Silica Plug* in *Supporting Information*). The collected solution was quenched with 1 M aqueous NaOH solution. A separatory funnel was used to separate out DCM and 1 M NaOH layers using ~10 mL of each. Separation was repeated 3 times and the organic layer was collected each time. Collected PMMA was dried under fan and then under vacuum at 70°C.

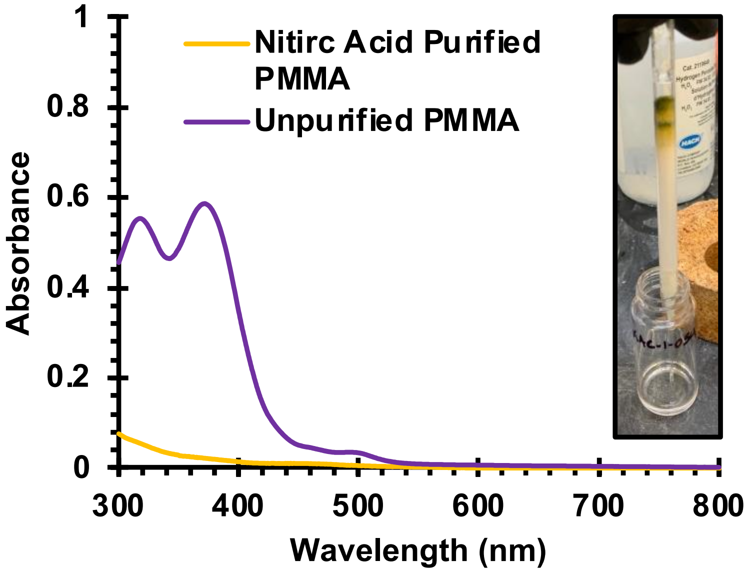

Figure S23. HNO_3 oxidized $\mathbf{1}^{\bullet+}$ and silica plug purification. A slight PC peak at 367 nm may be visible, but most PC appeared to be removed.

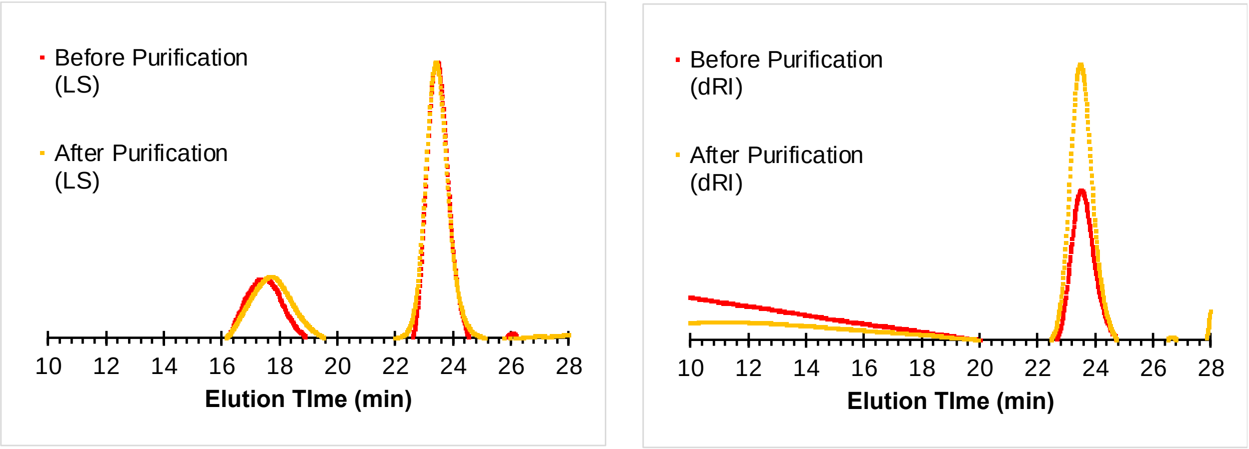

Figure S24. HNO_3 oxidized and silica plug purified PMMA before purification (red) and after purification (orange) using gel permeation chromatography light scattering (left) and dRI (right).

*<u>Br_2 · Dioxane Oxidation of **1** and Silica Plug Purification</u>*

Br_2 · Dioxane oxidation: 100.0 mg of PMMA containing residual **1** from O-ATRP was dissolved in 2.00 mL DCM. The solution was stirred until all PMMA dissolved. Br_2 · dioxane was dissolved in DCM to create a 1.00 mg/mL solution. Br_2 · dioxane (0.21 mL, 1 eq, 0.00085 mmol) was added the PMMA solution while stirring. Equivalence was determined by the estimation of PC remaining in PMMA (8.0 mg **1** • g^{-1} PMMA). The solution was allowed to stir 10 minutes and turned brown in color. Solution was then purified with a silica plug (See *Polymer Purification by Chemical Oxidation and Silica Plug* in *Supporting Information*).). No quenching was necessary. Collected PMMA was dried under fan and then under vacuum at 70°C.

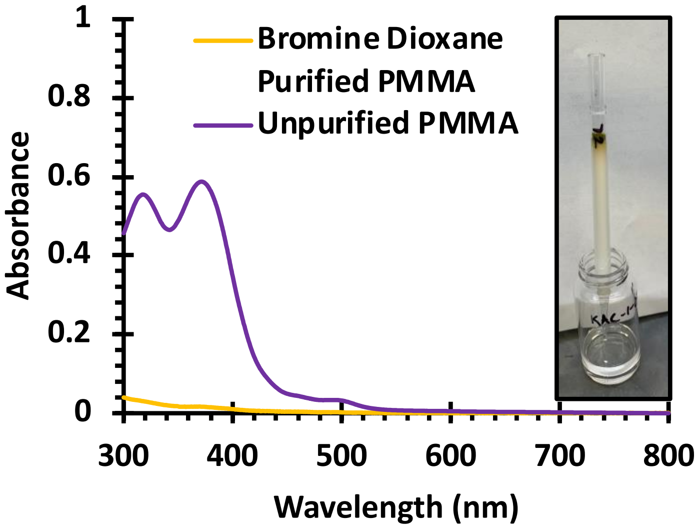

Figure S25. Br_2 · dioxane oxidized **1**$^{\bullet+}$ and silica plug purification. A slight PC peak at 367 nm may be visible, but most PC appeared to be removed.

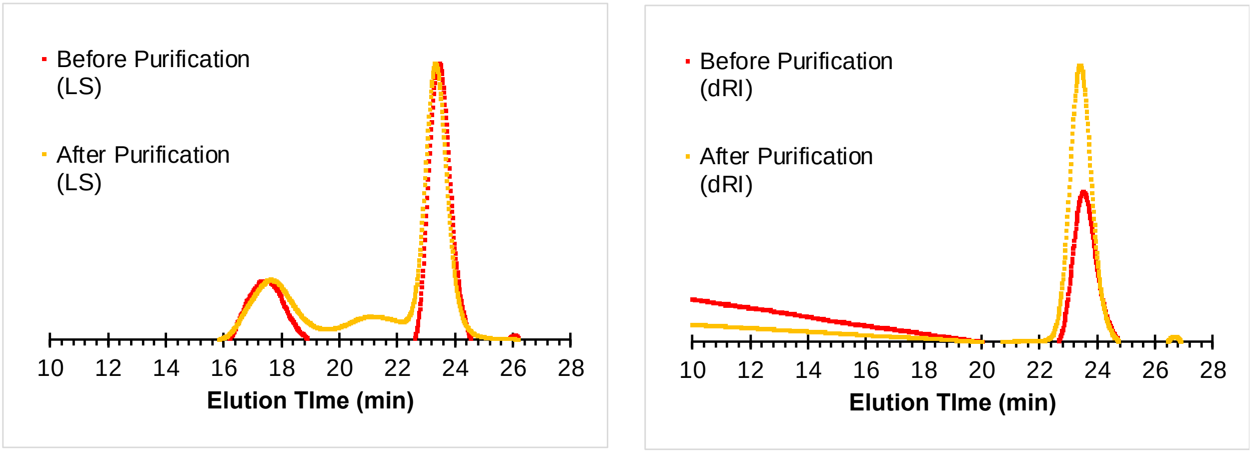

Figure S26. Br_2 · dioxane oxidized and silica plug purified PMMA before purification (red) and after purification (orange) using gel permeation chromatography light scattering (left) and dRI (right).

*NBS Oxidation of **1** and Silica Plug Purification*

NBS oxidation: 300.0 mg of PMMA containing residual **1** from O-ATRP was dissolved in 6.00 mL DCM. The solution was stirred until all PMMA dissolved. NBS was dissolved in DCM to create a 1.00 mg/mL solution. NBS (1.87 mL, 2 eq, 0.0102 mmol) was added to the PMMA solution while stirring. Equivalence was determined by the estimation of PC remaining in PMMA (8.0 mg **1** • g^{-1} PMMA). The solution was allowed to stir 10 minutes and turned green in color. Solution was then purified with a silica plug (See *Polymer Purification by Chemical Oxidation and Silica Plug* in *Supporting Information*). No quenching was necessary because all NBS appeared to be removed by the plug (Figure S28). Collected PMMA was dried under fan and then under vacuum at 70°C.

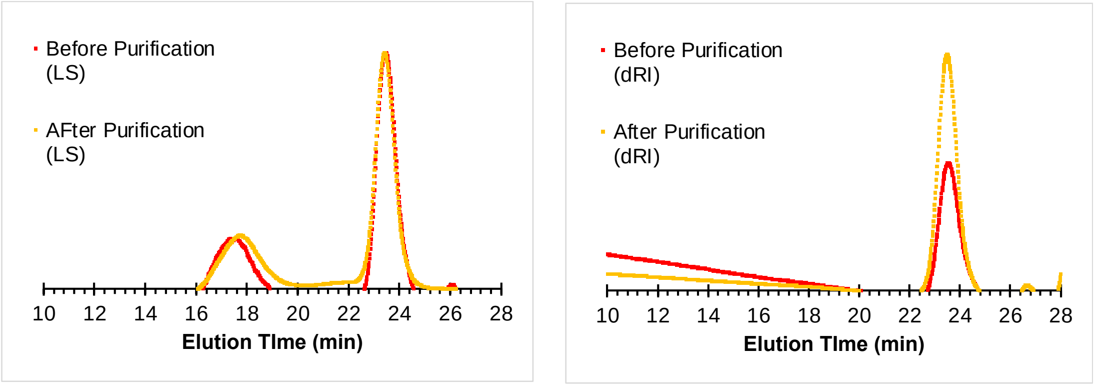

Figure S27. NBS oxidized and silica plug purified PMMA before purification (red) and after purification (orange) using gel permeation chromatography light scattering (left) and dRI (right).

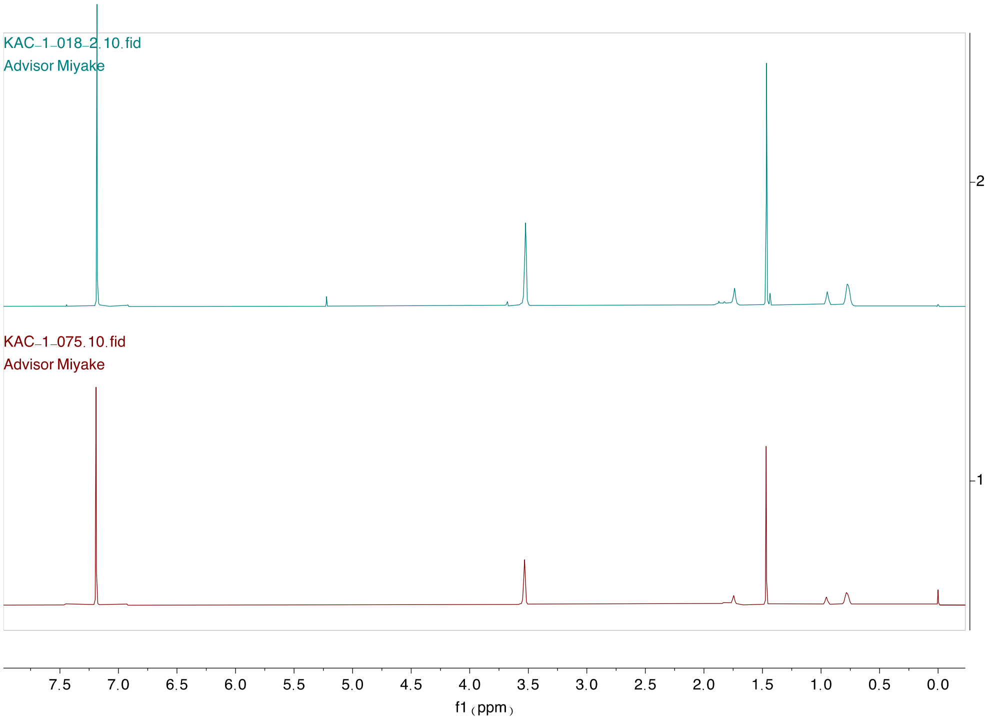

Figure S28. ^{1}H NMR comparing unpurified PMMA (blue) to NBS oxidized and silica plug purified PMMA (red).

Chain Extension

General Chain Extension Procedure

PMMA was synthesized via O-ATRP for all chain extensions. PC **1** was weighed into a scintillation vial and magnetic stir bars were added to each vial. Vials were brought into a nitrogen glove box. Under nitrogen, 1.00 mL ethyl acetate was added to each vial. Next, MMA (1.00 mL, 1000 eq, 9.35 mmol) and DBMM (17.9µL, 10 eq, 0.0935 mmol) were added in the dark. The vials were sealed and irradiated in a white LED light beaker for 24 hours while stirring. A fan blew over the beakers for temperature control.

After 24 hours, vials were removed from the glovebox and opened to dry for 3 days in a fume hood. For the purified PMMA samples, 300.0 mg PMMA was massed into a scint vial and dissolved in 10.00 mL DCM. The same oxidation procedures used in previous purifications were followed (See *Chemical Oxidants and Silica Plugs of* $1^{\bullet+}$ in *Supporting Information).* Oxidized $\mathbf{1}^{\cdot+}$ and PMMA was then run through an 800.0 mg silica plug and rinsed with 2.00 mL DCM. Chemical oxidants were appropriately quenched and then separated three times. Solutions were then left to dry under fan for 3 days.

GPC was used to determine the M_n of each PMMA sample, and exact masses of PMMA, **1**, and ethyl acetate (EtAc) are show below (Table S4). The isolated PMMA was then used as a macroinitiator (10 eq, 0.017 mmol) for each chain extension and was added to a scintillation vial with a magnetic stir bar. **1** (1 eq, 0.002 mmol) was added to a separate scintillation vial with a stir bar. All vials were brought into a nitrogen glove box. Half the volume of EtAc was added to the PMMA and stirred for 10 minutes. MMA (10000 eq, 17.0 mmol) was added to the vial containing **1** and stirred for 10 minutes. The **1** and MMA solution was then added to the EtAc + PMMA solution. The remaining EtAc was used to rinse any remaining PC into the PMMA solution. Solution was left to stir in the dark for 20 minutes until PMMA completely dissolved. The vials were then irradiated in a white LED light beaker for 24 hours with a fan for temperature control.

Table S4. Experimental Amounts of Reagents in Chain Extension

PMMA Purification Method	M_n (kDa)	Mass PMMA (mg)	Mass 1 (mg)	Volume MMA (mL)	Volume EtAc (mL)
**Unpurified**	8.86	200.0	1.1	2.46	1.00
**Br_2**	9.43	153.1	0.8	1.77	0.77
**I_2**	9.27	159.6	0.8	1.79	0.80
**NHO_3**	9.41	151.1	0.8	1.75	0.76
**Br_2· dioxane**	9.92	156.3	0.8	1.69	0.78
**NBS**	8.84	153.9	0.8	1.85	0.77

Unpurified PMMA

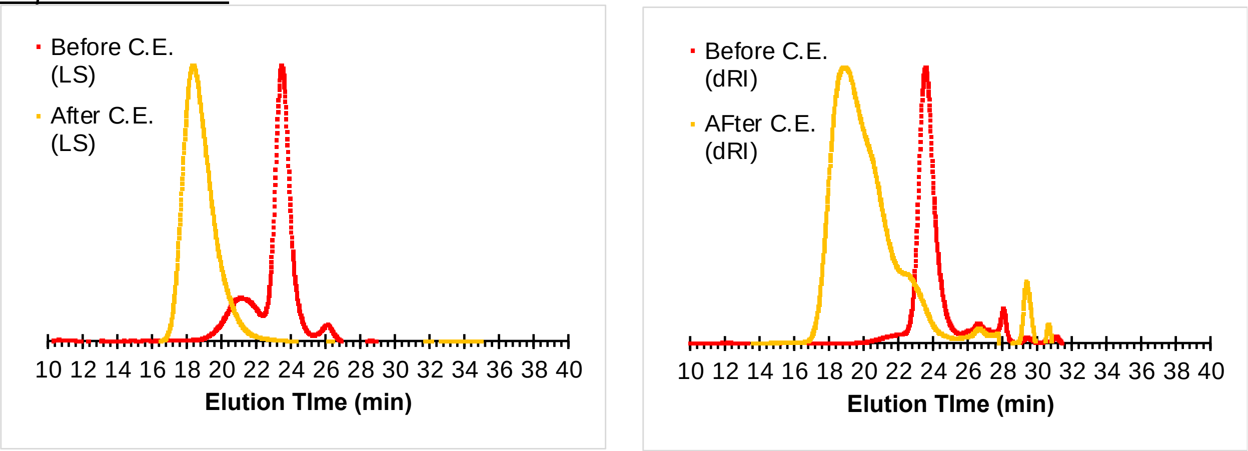

Figure S29. GPC trace of unpurified PMMA before chain extension (red) and of chain extended PMMA (orange). Both light scattering (left) and dRI (right) traces shown.

Br_2 Purified PMMA

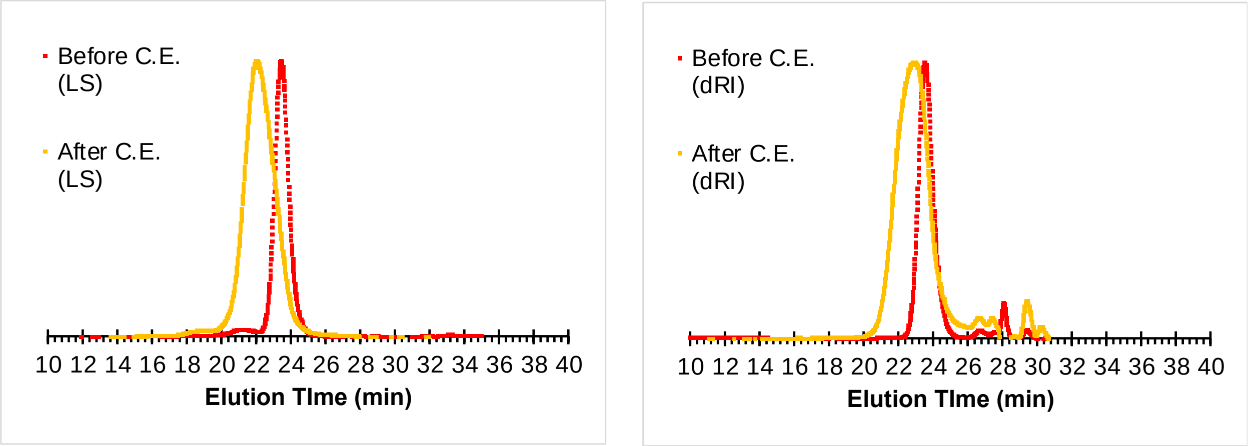

Figure S30. GPC trace of purified PMMA by Br_2 and silica plug (red) and of chain extended purified PMMA (orange). Both light scattering (left) and dRI (right) traces shown.

I_2 Purified PMMA

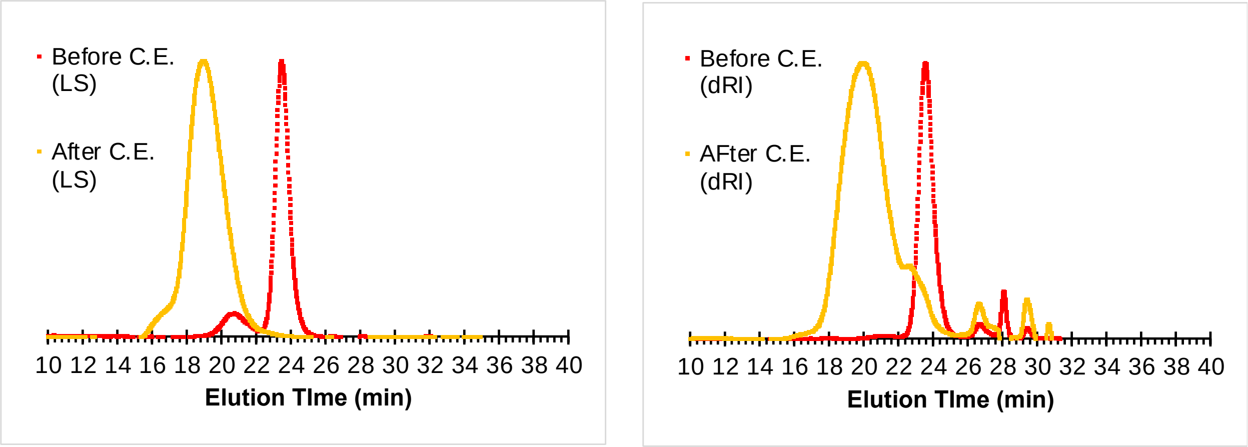

Figure S31. GPC trace of purified PMMA by I_2 and silica plug (red) and of chain extended purified PMMA (orange). Both light scattering (left) and dRI (right) traces shown.

HNO$_3$ Purified PMMA

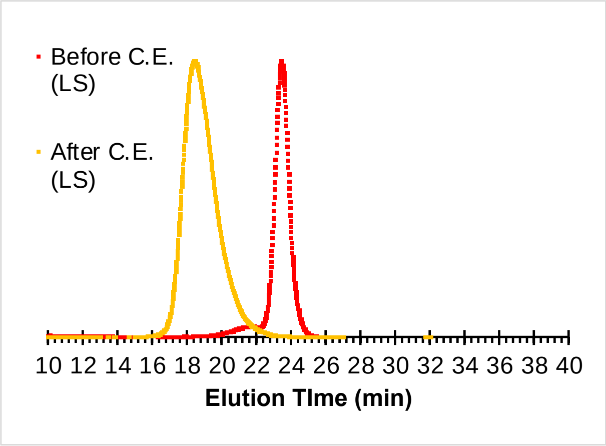

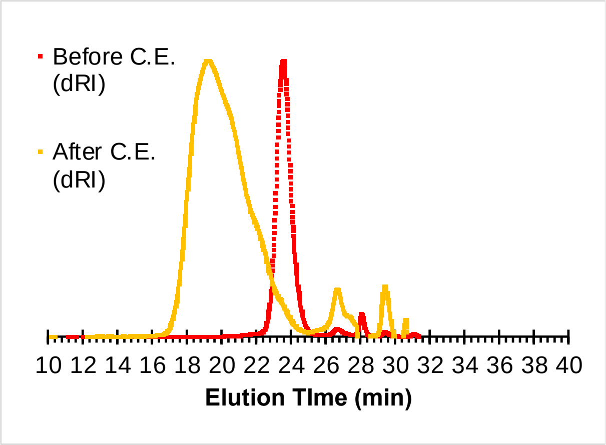

Figure S32. GPC trace of purified PMMA by HNO$_3$ and silica plug (red) and of chain extended purified PMMA (orange). Both light scattering (left) and dRI (right) traces shown.

Br$_2$ · dioxane Purified PMMA

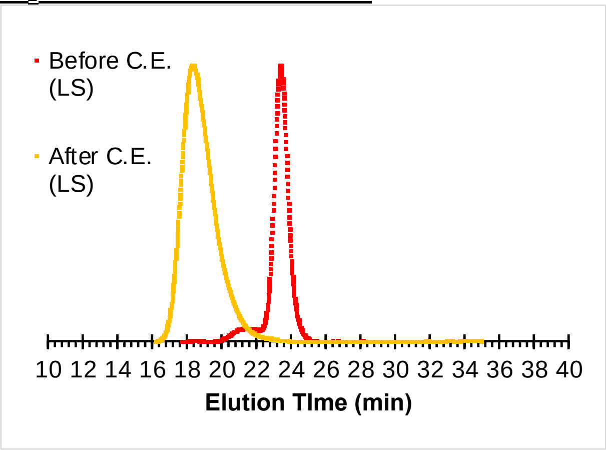

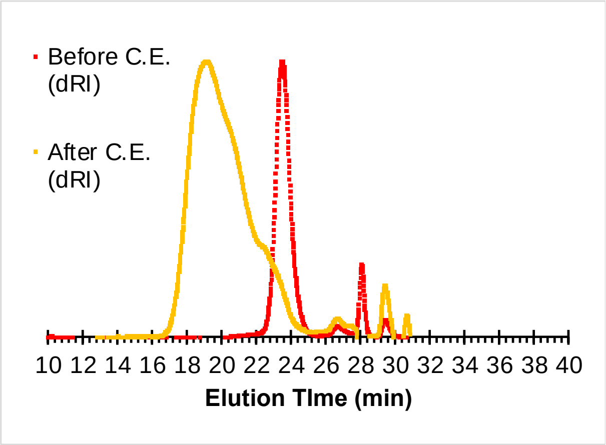

Figure S33. GPC trace of purified PMMA by Br$_2$ · dioxane and silica plug (red) and of chain extended purified PMMA (orange). Both light scattering (left) and dRI (right) traces shown.

NBS Purified PMMA

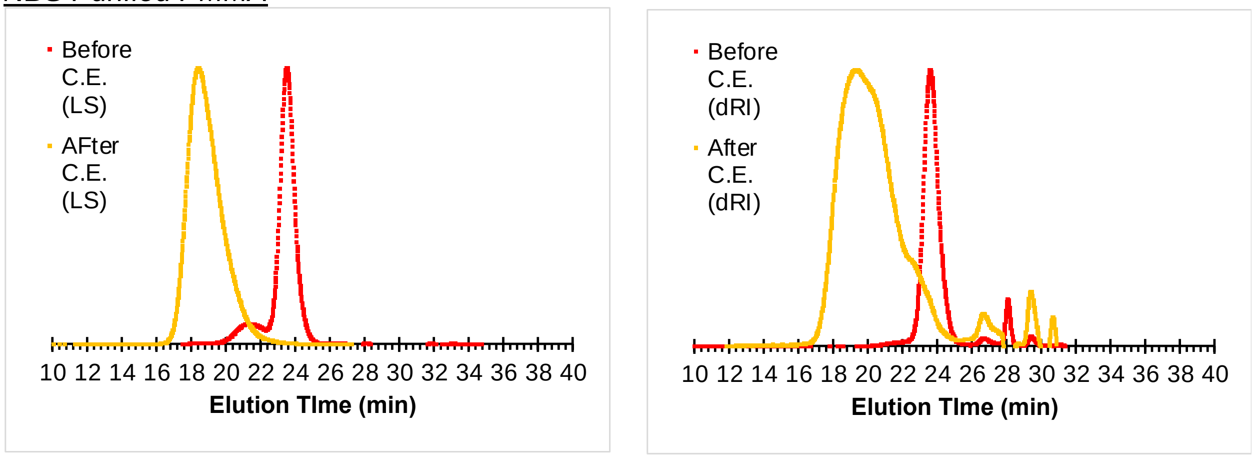

Figure S34. GPC trace of purified PMMA by NBS and silica plug (red) and of chain extended purified PMMA (orange). Both light scattering (left) and dRI (right) traces shown.

Table S5. Molecular weights of PMMA before and after chain extension

PMMA Purification Method	M_n Before (kDa)	M_n After (kDa)
**Unpurified**	8.86	98.9
**Br_2**	9.43	15.7
**I_2**	9.27	74.0
**NHO_3**	9.41	98.5
**Br_2· dioxane**	9.92	84.5
**NBS**	8.84	84.6

Oxidation Potential of Chemical Oxidants

Cyclic Voltammetry of NBS

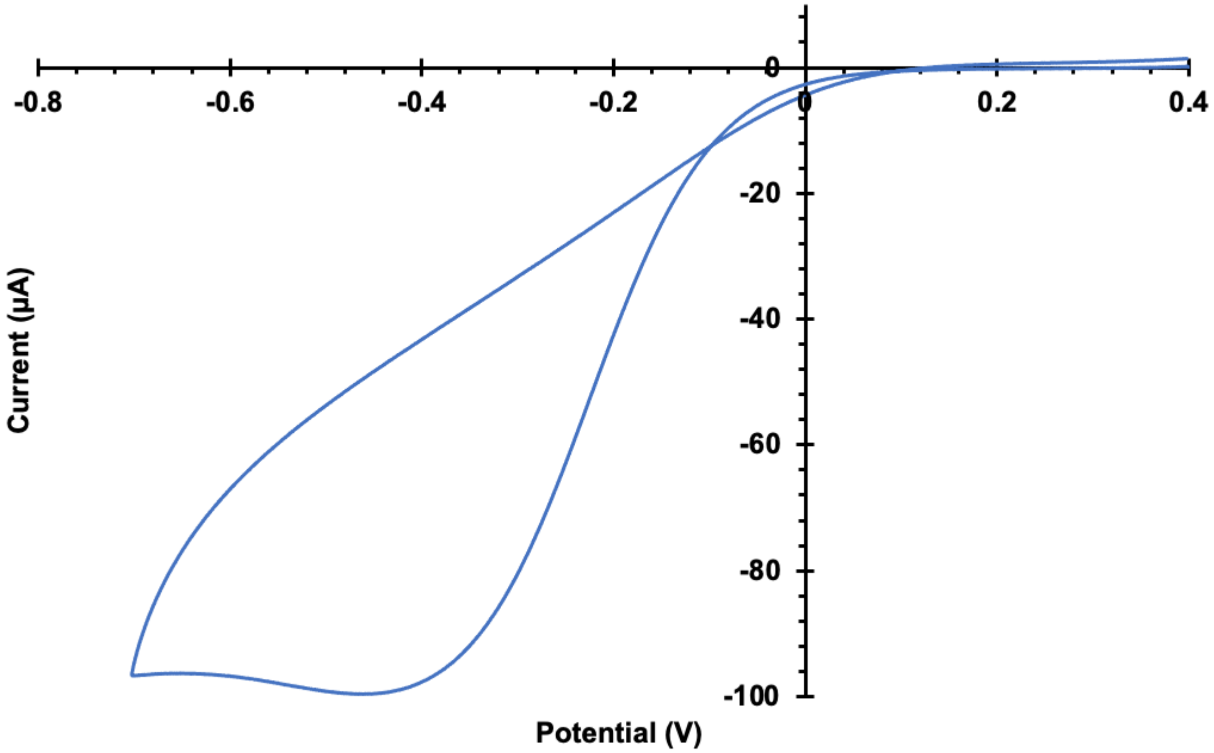

Figure S35. Cyclic Voltammogram of NBS vs Ag/AgNO$_3$ scanning from high to low potentials (0.4 V to -0.7 V). $E_{p/2}$ for reduction potential was determined to be -0.18 V vs Ag/AgNO$_3$ or 0.11 V vs SCE.

In the absence of a reversible peak and a clear $E_{1/2}$, $E_{p/2}$ can be used to estimate E^0.[9]

Oxidation Potentials of Additional Chemical Oxidants

Table S6. Standard Reduction Potentials for Chemical Oxidants Used in this Work

Oxidant	Reference	Solvent	E^0 (V vs SCE)
**Br_2**	[10]	acetonitrile	0.70
**I_2**	[10]	acetonitrile	0.26
**Br_2**	[11]	water	1.1
**HNO_3**	[11]	water	0.96
**I_2**	[11]	water	0.54

Purification of PMMA Synthesized with Other PCs

*O-ATRP Synthesis of PMMA with PCs **2-8***

Synthesis of PMMA: PC (0.0094 mmol, 1 eq) was weighed into a scintillation vial (Table S6) and brought into a nitrogen filled glovebox. DMAc (1.00 mL) was added to each vial. In the dark, MMA (1.00 mL, 9.35 mmol, 1000 eq) and DBMM (17.9 µL, 0.0935 mmol, 10 eq) were added to each vial. The reactions were then stirred and irradiated for 24 hours in UV or white LED beakers (Table S7). A desktop fan was used for temperature control. Light beakers had been warmed up (~30 minutes) before vials were added to ensure consistent and uniform irradiation. After 24 hours, an 0.1 mL aliquot of each vial was added to a solution of $CDCl_3$ with 250 ppm BHT and analyzed by ^{1}H NMR to evaluate percent conversion. The vials were then exposed to oxygen. PMMA was extracted from DMAc by adding 10.00 mL of deionized water and 10.00 mL of DCM and extracting the organic layer three times. Brine or additional DCM was added to help extraction. Samples were then dried under vacuum at 70°C for 72 hours.

Table S7. Details for O-ATRP with PCs 1-8

PC	Amount PC (mg)	Light Beaker
**1**	4.4	White LED
**2**	4.1	White LED
**3**	7.6	White LED
**4**	4.9	White LED
**5**	5.7	White LED
**6**	5.6	White LED
**7**	2.6	UV
**8**	5.1	UV

General PC Purification Scheme

NBS Oxidation with Silica Plug: For PCs **1-8**, the same purification method was followed as listed in *NBS Oxidation of **1** and Silica Plug Purification* in *Supporting Information.* The amount of PC per g PMMA was estimated by dividing the total amount of synthesized PMMA by the amount of PC used in O-ATRP. The estimated value of PC per gram of PMMA was used to determine amount of PC in 300.0 mg of PMMA and the amount of NBS to be added in a 2 to 1 equivalence with the PC.

UV-Vis Before and After Purification

UV-Vis Procedure: For each purified PMMA sample with PC **1-8**, the density of PMMA in DMAc was the same before and after purification for direct comparison. ~30.0 mg of PMMA was dissolved in 10.00 mL of DMAc and allowed to dissolve overnight while stirring. The same density before and after purification allowed absorbances to be compared to determine %PC removed (Table S8).

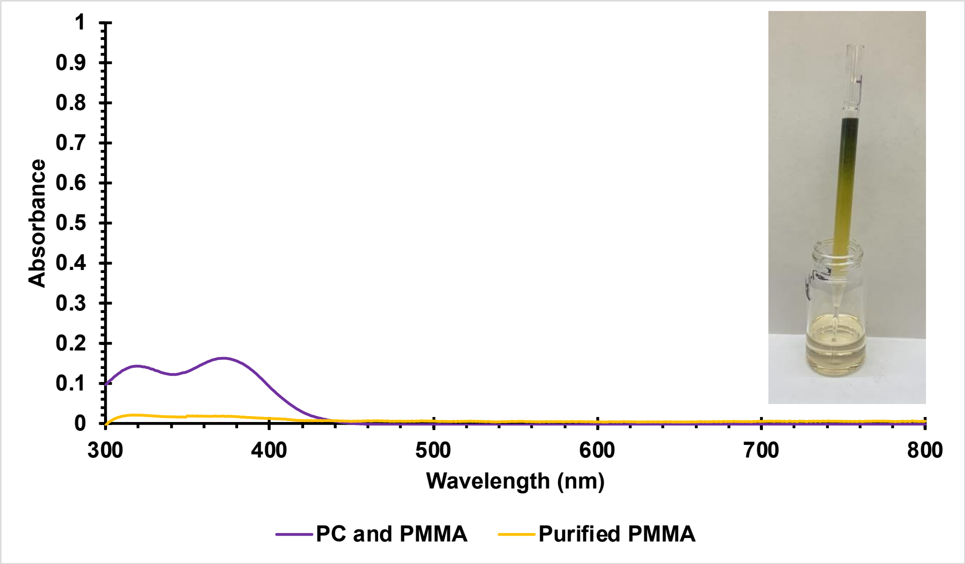

Figure S36. UV-Vis of PMMA before and after purification from PC **1** at 2.77 mg/mL density in DMAc.

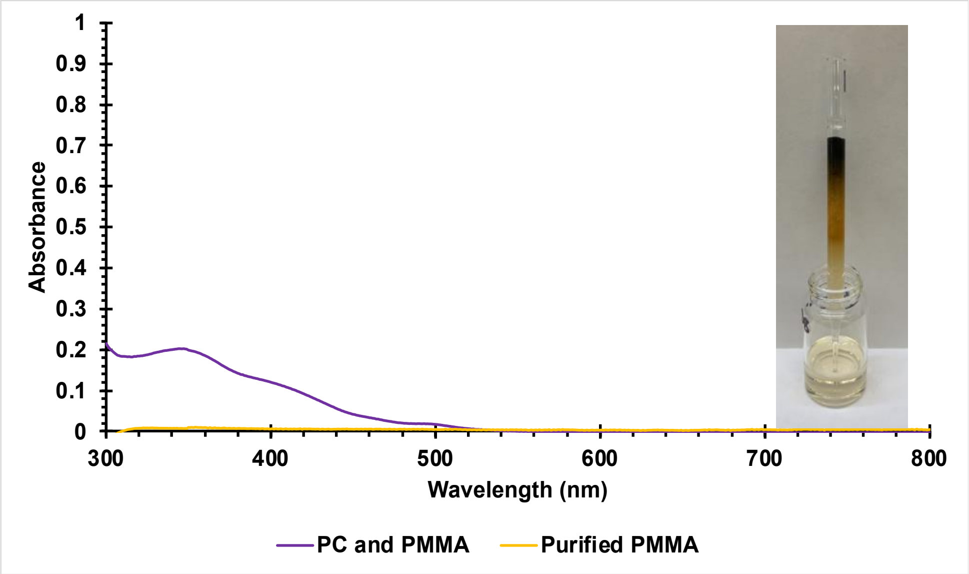

Figure S37. UV-Vis of PMMA before and after purification from PC **2** at 3.05 mg/mL density in DMAc.

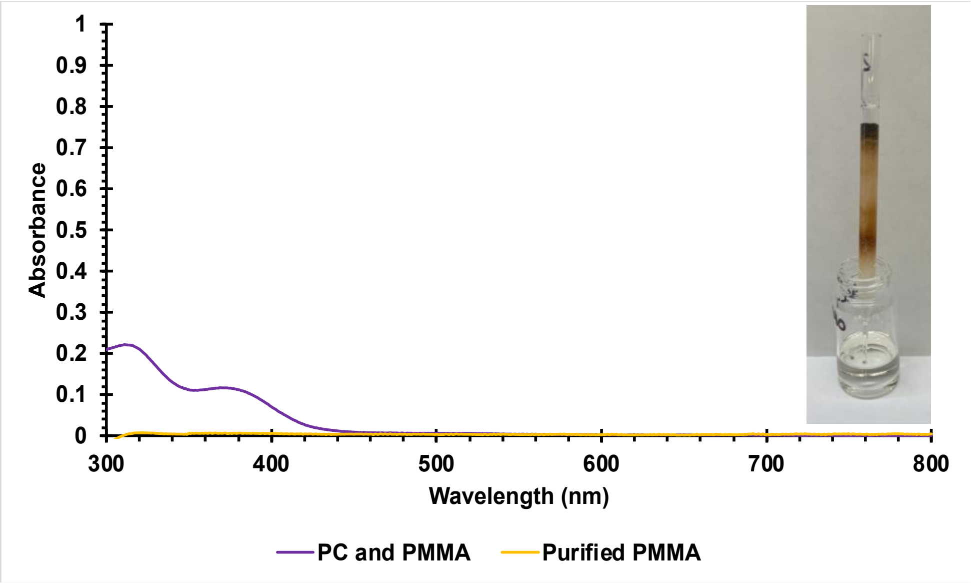

Figure S38. UV-Vis of PMMA before and after purification from PC **3** at 3.12 mg/mL density in DMAc.

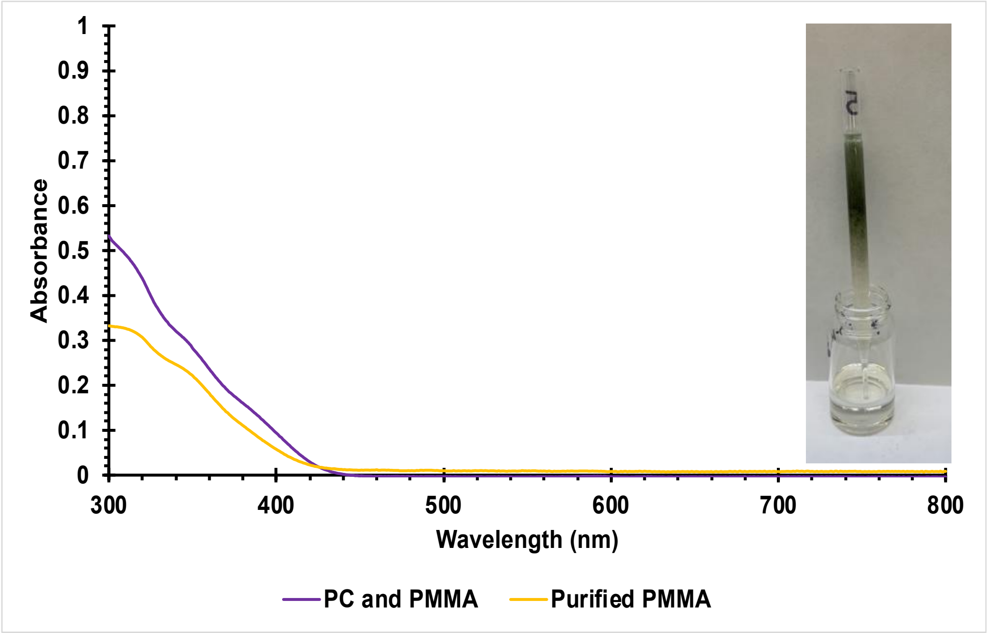

Figure S39. UV-Vis of PMMA before and after purification from PC **4** at 3.17 mg/mL density in DMAc.

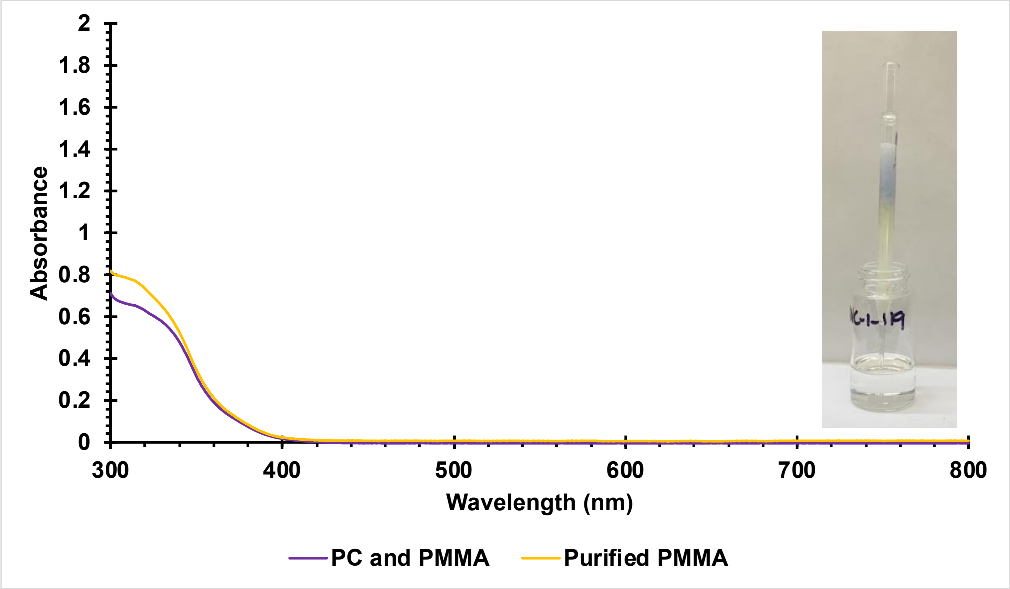

Figure S40. UV-Vis of PMMA before and after purification from PC **5** at 3.21 mg/mL density in DMAc.

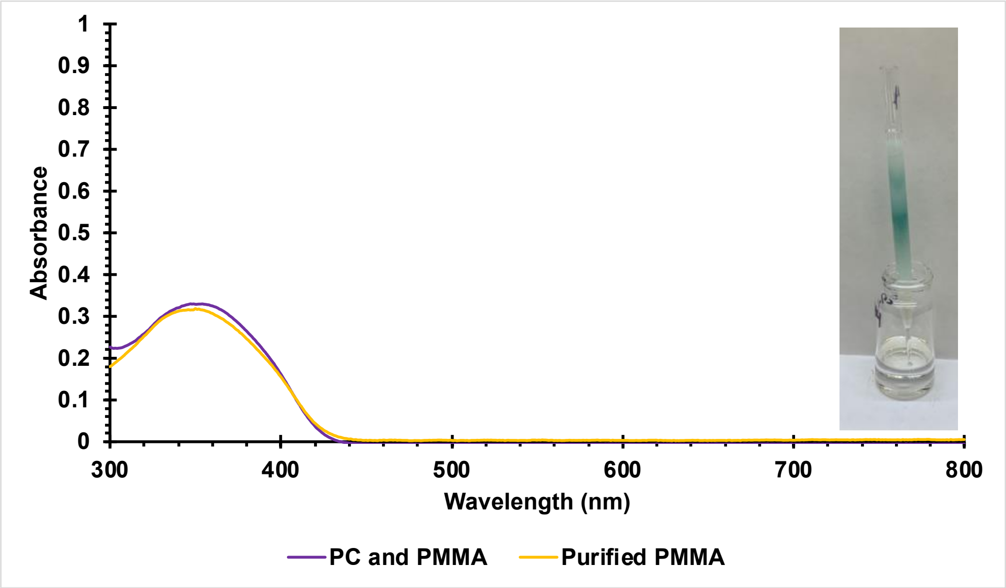

Figure S41. UV-Vis of PMMA before and after purification from PC **6** at 3.18 mg/mL density in DMAc.

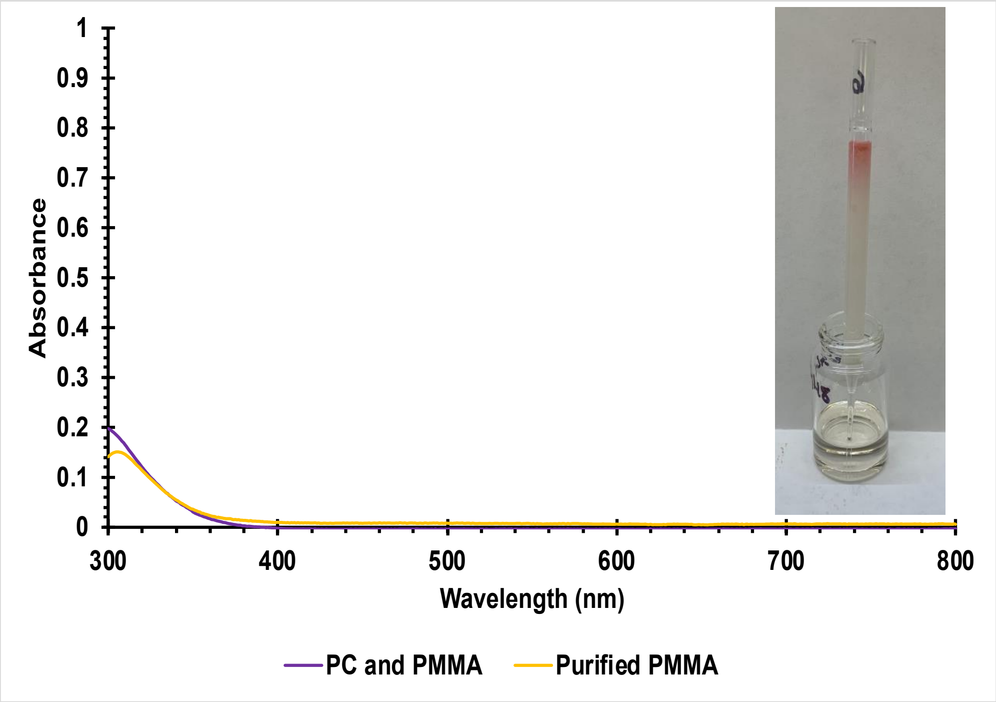

Figure S42. UV-Vis of PMMA before and after purification from PC **7** at 2.99 mg/mL density in DMAc.

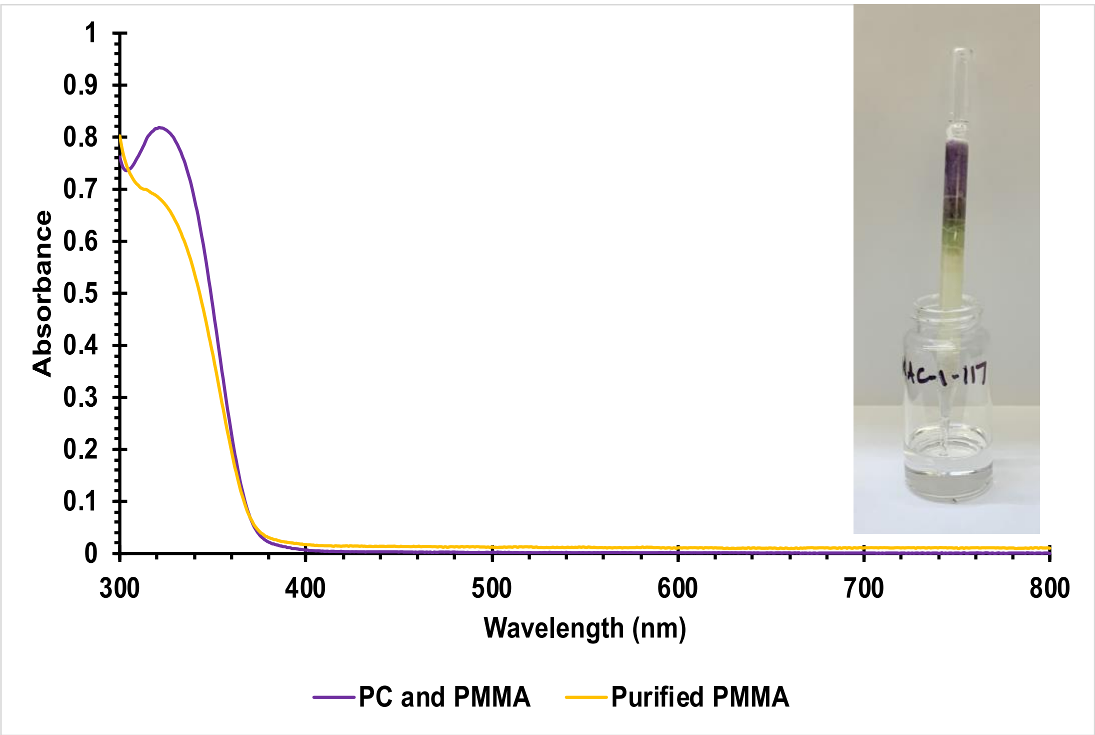

Figure S43. UV-Vis of PMMA before and after purification from PC **8** at 3.20 mg/mL density in DMAc.

%PC Remaining After Purification

Table S8. %PC remaining after purification for PCs 1-8

PC	$E_{1/2}$ (V vs SCE)	λ_{max} (nm)	Abs_{before}	Abs_{after}	%PC Removed[a]
**PhenN-2N**	0.19	345	0.202	0.008	96
**PhenN-PhCF$_3$**	0.29	367	0.161	0.018	89
**PhenN-PhCF$_3$-(M2BP)$_4$**	0.35	369	0.117	0.007	94
**PhenO-hex-2N**	0.61	351	0.279	0.218	22
**PhenO-1N-BiPh**	0.65	319	0.636	0.740	0
**PhenS-Ph**	0.68	305	0.184	0.150	18
**Acridine-1N-MeOPh**	0.71	321	0.819	0.683	17
**PhenO-2N-PhCF$_3$**	0.72	354	0.329	0.315	4

[a] Determined by %PC Removed = (Abs_{before} – Abs_{after}) / Abs_{before} * 100%

References

[1] J. C. Theriot, C. H. Lim, H. Yang, M. D. Ryan, C. B. Musgrave, G. M. Miyake, *Science* **2016**, *352*, 1082. DOI: 10.1126/science.aaf3935.
[2] D. A.Corbin, K. O. Puffer, K. A. Chism, J. P. Cole, J. C. Theriot, B. G. McCarthy, B. L. Buss, C. H. Lim, S. R. Lincoln, B. S. Newell, G. M. Miyake, *Macromolecules* **2021**, *54*, 4507. DOI: 10.1021/acs.macromol.1c00501.
[3] N. A. Swisher, D. A. Corbin, G. M. Miyake, *ACS Macro Lett.* **2021**, *10*, 453. DOI: 10.1021/acsmacrolett.1c00055.
[4] R. M. Pearson, C. H. Lim, B. G. McCarthy, C. B. Musgrave, G. M. Miyake, *J. Am. Chem. Soc.* **2016**, *138*, 11399. DOI: 10.1021/jacs.6b08068.
[5] B. G. McCarthy, R. M. Pearson, C. H. Lim, S. M. Sartor, N. H. Damrauer, G. M. Miyake, *J. Am. Chem. Soc.* **2018**, *140*, 508. DOI: 10.1021/jacs.7b12074.
[6] N. J. Treat, H. Sprafke, J. W. Kramer, P. G. Clark, B. E. Barton, J. R. de Alaniz, B. P. Fors, C. J. Hawker, *J. Am. Chem. Soc.* **2014**, *136*, 16096. DOI: 10.1021/ja510389m.
[7] B. L. Buss, C. H. Lim, G. M. Miyake, *Angew. Chem.* **2020**, *59*, 3209. DOI: 10.1002/anie.201910828.
[8] S. K. Chaudhuri, S. Roy, S. Bhar, *Beilstein J. Org. Chem.* **2012**, *8*, 323. DOI: 10.3762/bjoc.8.35.
[9] H. G. Roth, N. A. Romero, D. A. Nicewicz, *Synlett* **2016**, *27*, 714. DOI: 10.1055/s-0035-1561297.
[10] I. V. Nelson, R. T. Iwamoto, *J. Electroanal. Chem.* **1964**, *7*, 218.
[11] B. A. Averill, P. Eldredge, in *Principle of General Cehmistry: Principles, Patterns, and Applications,* Vol 1, Flat World Knowledge, 2011, Appendix E: Standard Reduction Potentials at 25°C.
